# Supplementary material for: Integrating nurses’ experiences with supporting behaviour change for cardiovascular prevention into a self-management internet platform in Finland and the Netherlands: a qualitative study
Source: BMJ Open. 2019 Jun 6;9(6):e023480. doi: 10.1136/bmjopen-2018-023480 (PMC6577411; doi:10.1136/bmjopen-2018-023480)
Supplement: Supplementary file 1 [file bmjopen-2018-023480supp001.pdf]

APPENDIX 1 to 'Integrating nurses' experiences with supporting behaviour change for cardiovascular prevention into a self-management internet-platform in Finland and the Netherlands: a qualitative study'

Appendix 1: COREQ checklist

No.	Item	Explanation	Reported on page no.
Domain 1:Research team and reflexivity			
1	Interviewers	NL: Pols AJ and Ligthart S<br>FI: Rosenberg A	Not reported in manuscript
2	Credentials of the researchers involved in data collection and analysis	NL: Beishuizen CRL: MD PhD-student; Rooskens F: BSc; Lighthart S: MD, PhD-student; Pols AJ: prof; Moll van Charante EP: MD PhD<br>FI: Akenine U: PhD-student; Barbera M: PhD; Rosenberg A: PhD-student	Not reported in manuscript
3	Occupation of the researchers involved in data collection and analysis	NL: Beishuizen CRL: PhD-student; Rooskens F: medical student; Ligthart S: general practitioner in training, PhD-student; Pols AJ: professor in medical ethics; Moll van Charante EP: postdoc researcher, general practitioner<br>FI: Akenine U: PhD-student, research nurse; Barbera M: postdoc researcher; Rosenberg A: PhD-student	Not reported in manuscript
4	Gender	All researchers are female, except for Moll van Charante EP, who is male	Not reported in manuscript
5	Experience and training	See main text	p5
6	Relationship established	NL: there was an indirect relationship between research team and the partcipants, because the nurses had been involved in a previous research project of the research team in which 2 researchers (Ligthart S and Moll van Charante EP) were also involved<br>FI: no previous relationship established	Not reported in manuscript
7	Partcipant knowledge of the	NL: participants knew the professional background of the moderators	Not reported in

	interviewer	FI: participants knew the professional background of the moderator	manuscript
8	Interviewer characteristics	NL: Pols AJ conducts qualitative research on the ethics of use of technology and eHealth in medical care. Ligthart S conducts quantitative and qualitative research on cardiovascular prevention in older people<br><br>FI: Rosenberg A conducts research on prevention of dementia	Not reported in manuscript
Domain 2: Study design			
9	Methodological information and theory	See main text	P5 and p6
10	Sampling	See main text	P5
11	Method of approach	See main text	P5
12	Sample size	See main text	P5
13	Non-participation	See main text	P5
14	Setting of data collection	See main text	P5
15	Presence of non-participants	NL: Eric Moll- van Charante and Pim Happel were present as non-participating audience<br><br>FI: no others were present	Not reported in manuscript
16	Description of sample	See main text	P5 and p6
17	Interview guide	See main text	P6
18	Repeat interviews	Not performed	
19	Audio/visual recording	See main text	P6
20	Field notes	See main text	P6
21	Duration	See main text	P6

22	Data saturation	See main text	P7 and P15
23	Transcripts returned	Not performed	
Domain 3: analysis and findings			
24	Number of data coders	NL: 2<br>FI: 2	P6
25	Description of the coding tree	See main text (figure 1)	p14
26	Derivation of themes	See main text	P6
27	Software	No special qualitative software was used	
28	Participant checking	See main text	P7 and p15
29	Quotations presented	See main text	P8– p13
30	Data and findings consistent	See main text	P8 – p13
31	Clarity of major themes	See main text and figure 1	P8-p14
32	Clarity of minor themes	Within the groups, the Dutch and Finnish nurses shared opinions and experiences on most topics. Between the Dutch and Finnish groups, some interesting differences in opinions and experiences were identified. We choose therefore to focus on these differences when presenting our results, but not on diverse cases within the groups.<br><br>Minor themes were not discussed due to word limits	Not reported in manuscript
