# Supplementary material for: Integrating nurses’ experiences with supporting behaviour change for cardiovascular prevention into a self-management internet platform in Finland and the Netherlands: a qualitative study
Source: BMJ Open. 2019 Jun 6;9(6):e023480. doi: 10.1136/bmjopen-2018-023480 (PMC6577411; doi:10.1136/bmjopen-2018-023480)
Supplement: Supplementary file 2 [file bmjopen-2018-023480supp002.pdf]

APPENDIX 2 to 'Integrating nurses' experiences with supporting behaviour change for cardiovascular prevention into a self-management internet-platform in Finland and the Netherlands: a qualitative study'

Appendix 2: Topic list

**FOCUS GROUP nurses part ONE cardiovascular prevention**		
**Overview part one**	**Topic (possible items)**	**Possible questions**
Aim of the meeting is to learn from the nurses' experiences in cardiovascular prevention: what are best practices to stimulate behaviour change, what is their attitude towards a prevention program for older people via the internet, how would they support people in lifestyle change through the internet?		
	**CVD prevention**	
	Activities on CVD prevention	Do you currently conduct activities on CVD prevention?
	Experiences / lessons learned	What are your experiences and lessons learned, especially regarding giving 'medical' guidance versus giving lifestyle guidance and regarding behavior change?
	**Dementia prevention**	In the last decade, we got more and more indications from neurological research that risk factors for cardiovascular disease are also risk factors for dementia. So, possibly, reducing cardiovascular risk may also postpone or prevent dementia.
	Attitude towards dementia prevention	What is your attitude / your ideas regarding dementia prevention?
	Awareness of dementia risk and compliance to lifestyle change	Most people are not yet aware of the association between CV risk and dementia, but do seem to be very afraid of dementia. Do you think that more awareness would enhance compliance/adherence to lifestyle change? What are your ideas on this?
	**Relationship with participants and regular healthcare system**	
	Guiding lifestyle change	Which factors could contribute to good guidance of lifestyle change?
	Relationship with patient	Which factors could contribute to a good relationship with your patient?
	Relation with GP and regular practice assistant	How should the platform coach link with the patients' GP and regular practice assistant/nurse (the regular healthcare system)?

	Attitude towards internet intervention	What is your attitude towards a prevention program via the internet (with support from a coach)?
		How should the platform coach link with the patients' GP and regular practice assistant/nurse (the regular healthcare system)?
**FOCUS GROUP nurses part TWO The platform**		
Assistant moderator explains about HATICE and platform by showing the powerpoint with screen shots of the platform		
	**Information required for support**	Imagine yourself being a coach using the internet-platform:
		Which kind of information regarding the participants do you need to be able to support them?
	**Role and responsibilities**	
	Responsibility goal setting	Who is responsible for goal setting? (capability of patients)
	Role in lifestyle groups	How do you see your role in creating lifestyle groups and how can participation be encouraged?
	**Interaction with participants**	
	Experience with motivational interviewing	Do you have experience with motivational interviewing, how could this technique be used by the coach?
	Frequency of contact	How often would you like to have contact with your patient?
	Mode of communication	Which mode of communication do you prefer? (phone, skype +/- webcam, email, chat…)
	Network of support	What should be the role of the participant network of support in lifestyle change and how can this network be engaged?
	Alerts / reminders	Do you like to receive automatic alerts/reminders when patients have alarming values or did not log-on?
Assistant moderator and moderator verify key messages from focus group		
Moderator thanks nurses		
