# Supplementary material for: Integrating nurses’ experiences with supporting behaviour change for cardiovascular prevention into a self-management internet platform in Finland and the Netherlands: a qualitative study
Source: BMJ Open. 2019 Jun 6;9(6):e023480. doi: 10.1136/bmjopen-2018-023480 (PMC6577411; doi:10.1136/bmjopen-2018-023480)
Supplement: Author's manuscript [file bmjopen-2018-023480.draft_revisions.pdf]

BMJ Open

BMJ Open is committed to open peer review. As part of this commitment we make the peer review history of every article we publish publicly available.

When an article is published we post the peer reviewers' comments and the authors' responses online. We also post the versions of the paper that were used during peer review. These are the versions that the peer review comments apply to.

The versions of the paper that follow are the versions that were submitted during the peer review process. They are not the versions of record or the final published versions. They should not be cited or distributed as the published version of this manuscript.

BMJ Open is an open access journal and the full, final, typeset and author-corrected version of record of the manuscript is available on our site with no access controls, subscription charges or pay-per-view fees (http://bmjopen.bmj.com).

If you have any questions on BMJ Open's open peer review process please email info.bmjopen@bmj.com

BMJ Open

Integrating nurses' experiences with supporting behaviour change for cardiovascular prevention into a self-management internet-platform in Finland and the Netherlands: a qualitative study

Journal:	*BMJ Open*
Manuscript ID	bmjopen-2018-023480
Article Type:	Research
Date Submitted by the Author:	27-May-2018

Complete List of Authors:	Beishuizen, Cathrien; Academic Medical Center, General Practice<br>Akenine, Ulrika; Karolinska Institutet and Karolinska University Hospital, Division of Clinical Geriatrics , Department of Neurobiology, Care Sciences and Society<br>Barbera, Mariagnese; University of Eastern Finland, Clinical Medicine/Neurology<br>Rosenberg, Anna; University of Eastern Finland, Clinical Medicine/Neurology<br>Fallah Pour, Mandana; Karolinska Institutet, Division of Occupational Therapy, Department of Neurobiology, Care Sciences and Society<br>Richard, Edo; Academic Medical Centre, University of Amsterdam, Neurology; Radboud University Medical Center, Department of Neurology, Donders Institute for Brain, Cognition and Behaviour<br>Soininen, Hilkka; University of Eastern Finland, Clinical Medicine/Neurology<br>Mangialasche, Francesca; Karolinska Institutet, Division of Geriatric Epidemiology<br>Kivipelto, Miia; Karolinska Institutet, Division of Geriatric Epidemiology; University of Eastern Finland, Clinical Medicine/Neurology<br>Pols, A.; AMC, Department of General Practice, Section of Medical Ethics,<br>Moll van Charante, Eric; Academic Medical Center, General Practice

Keywords:	QUALITATIVE RESEARCH, PRIMARY CARE, Nurse-led care, eHealth, Cardiovascular prevention, Dementia prevention

SCHOLARONE™
Manuscripts

Integrating nurses' experiences with supporting behaviour change for cardiovascular prevention into a self-management internet-platform in Finland and the Netherlands: a qualitative study

Authors

Beishuizen CRL[1*], Akenine U[2], Barbera M[3], Rosenberg A[3], Fallahpour M[4], Richard E[5,6], Soininen H[3,7], Mangialasche F[8], Kivipelto M[2, 3,9], Pols AJ[10] and Moll van Charante EP[1]

*corresponding author

address: Department of General Practice
Academic Medical Centre/University of Amsterdam
Meibergdreef 9, 1100 DD
Amsterdam, Netherlands

email: c.r.beishuizen@amc.uva.nl

phone: +31 6 16359453

Affiliations

[1] Department of General Practice, Academic Medical Centre, University of Amsterdam Amsterdam, The Netherlands

[2] Division of Clinical Geriatrics, Department of Neurobiology, Care Sciences and Society, Karolinska Institutet, and Karolinska University Hospital, Stockholm, Sweden

[3] Institute of Clinical Medicine/Neurology, University of Eastern Finland, Kuopio, Finland

[4] Department of Neurobiology Care Sciences and Society, Division of Occupational Therapy, Karolinska Institutet, Huddinge, Sweden

[5] Department of Neurology, Academic Medical Centre, University of Amsterdam, Amsterdam, The Netherlands

[6] Department of Neurology, Donders Institute for Brain, Cognition and Behaviour, Radboud University Medical Center, Nijmegen, The Netherlands

[7] Neurocenter, Neurology, Kuopio University Hospital, Kuopio, Finland.

[8] Aging Research Center, Karolinska Institutet and Stockholm University, Stockholm, Sweden

[9] Chronic Disease Prevention Unit, National Institute for Health and Welfare, Helsinki,, Finland

[10] Section of Medical Ethics, Department of General Practice, Academic Medical Centre, University of Amsterdam, Amsterdam, The Netherlands

Word count

Total text (main text and quotations): 4,254

For peer review only
For peer review only - http://bmjopen.bmj.com/site/about/guidelines.xhtml

Abstract

Objectives: Global ageing is linked to an increased burden of cardiovascular disease (CVD) and dementia, which calls for better prevention strategies. Self-management and eHealth applications are regarded promising strategies to support prevention. The aim of this study was to explore primary care nurses' experiences with behaviour change guidance for cardiovascular (CV) prevention to learn how to optimally integrate these into an internet-platform with coaching for cardiovascular self-management.

Design: Qualitative focus group study in Finland and the Netherlands.. Discussions were audiotaped and transcribed. Data were thematically analysed following grounded theory.

Setting: Dutch and Finnish primary care settings.

Participants: Six Finnish and seven Dutch nurses experienced in CV prevention

Results: Finnish and Dutch nurses expressed similar experiences with supporting behaviour change for CV prevention but used different practical approaches, which was reflected in their recommendations for online-support. Both groups emphasised that online-support should be combined with human-support and integrated in regular care. Finnish nurses had more confidence in patient self-management and remote communication than Dutch nurses, who emphasised the importance of face-to-face contact and preferred to keep the control on medical aspects of prevention.

Conclusions: Differences in CV prevention support of Dutch and Finnish nurses appear to reflect their local healthcare practices, which should be taken into account when designing internet-platforms for health self-management. Including cognitive health as a goal of CV prevention might stimulate people's motivation for health behaviour change.

Keywords: cardiovascular prevention, dementia prevention, behaviour change, eHealth, primary care, nurse-led care, qualitative research

Strengths and limitations of this study

- We performed an international qualitative study and made a direct comparison of the experiences and health care practices of Finnish and Dutch primary care nurses within their healthcare contexts.
- This study provides directions for innovative forms of preventive healthcare, by having experienced nurses translate their current best practices of health behaviour change support towards an online health-support setting.
- Our findings might be limited to health care settings that resemble Finnish and Dutch cardiovascular preventive care organisation.

For peer review only

Manuscript

INTRODUCTION

Global ageing places an increasing demand on healthcare systems, partially due to the absolute rise in cardiovascular disease (CVD) and dementia cases [1 2]. As these disorders share a number of risk factors, effective cardiovascular (CV) prevention could also lead to the prevention of dementia [3-6]. CV prevention requires health behaviour change, the process of "initiating and maintaining behaviours that reduce health risks and control existent chronic disease"[7]. In CV prevention, core behaviours consist of a healthy lifestyle (healthy diet, sufficient physical activity and non-smoking) and adherence to medication. Although the processes behind supporting health behaviour change have been theorised extensively[8-12], putting them into practice remains a challenge[13 14] and novel, more effective, approaches are needed[15]. Two strategies of current interest are self-management and eHealth. In self-management, the individual, instead of the healthcare professional, takes the lead in the management of his/her risk factors and adherence, and therefore in behaviour change[16 17]. eHealth applications can easily support self-management and are attractive because of their wide reach, low-cost and suitability for health education[18 19]. Although researchers and policymakers have high expectations of eHealth and self-management, little is known of how self-management and behaviour change are best stimulated and maintained online.

This project is part of the Healthy Ageing Through Internet Counselling in the Elderly (HATICE) study, which includes a European randomised controlled trial testing a coach-supported internet-platform for self-management of cardiovascular risk factors in older people to prevent CVD and cognitive decline [20]. In an international focus group study, we aimed to explore (1) nurses' experiences and practices with behaviour change guidance for cardiovascular prevention, including the potential for dementia prevention, and (2) how to integrate their practices into a coach-supported internet-platform (the online-support setting). This study took place in Finland and the Netherlands, two of the three countries where the HATICE-study is ongoing. Since the HATICE project aims to develop an internet-platform that is implementable across all European healthcare systems, we also explored the influence of local healthcare practices.

For peer review only - http://bmjopen.bmj.com/site/about/guidelines.xhtml

METHODS

We performed an international qualitative focus group study following grounded theory[21 22]. The COREQ-checklist is included for complete information on methodology (**Appendix 1**)[23].

Participants and setting

Finnish and Dutch primary care nurses experienced in cardiovascular preventive care were eligible for this study and selective purposive samples were obtained, following one of the grounded theory methods; i.e. studying a health care practice by consulting field experts [24]. In Finland, we recruited occupational healthcare nurses because of their important role in preventive CV care. Nurses working in a semi-private healthcare centre in Kuopio (Eastern Finland) were invited and six female nurses consented to participate. Being occupational health nurses they cared mostly for patients in the working age. In the Netherlands, we recruited primary care nurses experienced in cardiovascular risk management. A group of 32 nurses working in general practices in two urban areas in the centre of the Netherlands was invited and seven female nurses consented to participate. The Dutch participating nurses cared for patients of all ages. **Table 1** contains further characteristics.

Table 1. Characteristics of the participating Finnish and Dutch nurses

Nr	Country*	Age	Education	Experience (years)	Typ of CVD prevention	Additional expertise	Internet use at work
**1**	FI	55	occupational health nurse	33	prim/sec prev	psychology and stress	email, guideline use, referral, patient contact
**2**	FI	42	occupational health nurse	20	prim/sec prev	none	email, guideline use, referral
**3**	FI	25	occupational health nurse	2	prim/sec prev	none	email, guideline use, referral, patient contact
**4**	FI	45	occupational health nurse	15	prim/sec prev	mental health	email, guideline use, referral, patient contact
**5**	FI	49	occupational health nurse	23	prim/sec prev	none	email, guideline use, referral, patient contact
**6**	FI	60	occupational health nurse	35	prim/sec prev	mental health	guideline use, patient contact
**1**	NL	43	general nurse, practice nurse[a]	7	prim/sec prev	DM, COPD, mental health, elderly	email, guideline use, referral, patient contact
**2**	NL	49	practice nurse	10	prim/sec prev	DM, COPD, older people	email, guideline use, referral, patient contact
**3**	NL	51	practice nurse	3	prim prev	DM, COPD, older people	email, guideline use, referral, patient contact
**4**	NL	53	general nurse, practice nurse	6	prim/sec prev	DM, COPD, older people	email, guideline use, referral
**5**	NL	42	practice nurse	4	sec prev	DM, COPD, older people	email, guideline use, referral, patient contact
**6**	NL	45	general nurse, practice nurse[b]	11	prim/sec prev	DM, COPD, older people	email, guideline use, referral, patient contact
**7**	NL	65	general nurse, practice nurse	11	prim/sec prev	none	email, guideline use, referral, patient contact

For peer review only - http://bmjopen.bmj.com/site/about/guidelines.xhtml

*abbreviations: CVD = cardiovascular disease, FI = Finland, prim = primary, sec = secondary, prev = prevention, NL = the Netherlands, DM = diabetes mellitus, COPD = chronic obstructive pulmonary disease

[a] practice nurse: received specific nursing training to work in the general practice
[b] general nurse: received general nursing training to work as a general nurse in the hospital

The study was presented to the medical ethics committee of the Academic Medical Centre in the Netherlands and a waiver was provided. In Finland, application for ethical approval nor a waiver were required. All participants provided written informed consent.

Data collection

We conducted one focus group in the Netherlands (autumn 2013) and one in Finland (December 2015). In each country, an experienced focus group moderator chaired the sessions, while an assistant-moderator noted non-verbal communication and summarised the discussions. The discussion was conduction using a topic list as reference (**Box 1** and **Appendix 2**). After the Dutch session, the topic list was refined for the Finnish focus group. The moderators asked open-ended questions, following grounded theory to inductively approach the data. Both moderators first discussed the nurses' activities in cardiovascular prevention and how they supported their patients in the process of behaviour change. The Finnish moderator also discussed the nurses' experiences on prevention of dementia. In the second part, the HATICE internet-platform was presented (**Box 2**, a full description of the platform is reported elsewhere [25]) and the nurses were asked how they would optimally support their patients in an online setting. Both sessions lasted approximately two hours. The discussions were audio-recorded and transcribed.

Box 1. Main topics discussed

Box 2. Key features of the HATICE internet-platform with coaching

Coding and analysis

In each country, two researchers coded and thematically analysed the transcripts following grounded theory [21 22]. Themes were derived inductively from the data and were not hypothesised beforehand. Open coding and identification of initial themes was first performed by the two researchers independently. Thereafter, codes and themes were compared. Dissimilarities were discussed until consensus was reached. Initial theme structure was then discussed with the senior researchers involved. In Finland, since the researchers were not Finnish native speakers, the transcript was translated into English and cross-checked by the Finnish focus group moderator, who was a Finnish native fluent English speaker. In this way, the complete analysis of the Finnish data could be performed in English. After the initial analysis performed locally, themes and corresponding

For peer review only - http://bmjopen.bmj.com/site/about/guidelines.xhtml

quotations of the Dutch sessions were also translated into English. The two research teams then had two meetings to discuss the structure of main themes and categories. The analysis-phase[21 22] was an iterative process, during which the researchers of both teams repeatedly returned to their data-files to add, merge and refine themes, until a definite theme structure was agreed on by all authors. During the iterative analysis-phase, the researchers discussed the themes and alternatives and it was proposed that the local health care context was of influence on the differences found between caring styles of the two groups of nurses. Therefore, the research teams introduced their local health care systems (**Box 3**) to each other and these insights were used in further interpretation of the findings. A summary of the final conclusions was returned to the participants for feedback.

Box 3. The Finnish and Dutch primary care systems

Patient and public involvement

Patients and public were not involved in the design of this study.

RESULTS

We present our findings in two sections: 1) Nurses experiences and practices with supporting the process of behaviour change for cardiovascular prevention, including the potential for dementia prevention, and 2) Their suggestions on how to integrate their experiences in an online-support setting.

Part 1: Nurses experiences and practices with supporting the process of behaviour change for cardiovascular prevention

Preconditions for behaviour change guidance

We identified three main themes, that both the Finnish and Dutch nurses regarded as preconditions for behaviour change guidance in their patients: establishing a relation of trust, awareness and expectation management and appropriate timing and monitoring. Both groups of nurses explained what skills they used to realise these preconditions, showing subtle differences between the groups.

Establishing a relationship of trust

According to both the Finnish and Dutch nurses, the basis of behaviour change support lied in establishing a relationship of trust with the patient: developing a good nurse-patient relationship in which the individual felt at ease and respected and comfortable enough to open up about lifestyle and behaviour issues:

For peer review only - http://bmjopen.bmj.com/site/about/guidelines.xhtml

"For lifestyle change, for prevention, a relationship based on mutual trust is pivotal. It is good to have a many years' standing contact with people. Then you know what is going on in someone's life and in that, some kind of trust will grow that people really start believing what you are saying to them. And then, over time, people will start practising healthy behaviours that maybe they had no intention to follow, in the beginning" (Dutch nurse 1)

Skills the nurses used to stimulate trust to grow, were personalising and tailoring their support to each patient:

"And you need to get a good picture of the situation, so that you don't give the same information to everyone. That's of no use. You need to think what the central issues are for this patient. What are the things he or she seems to have resources for? What are the goals that the client sets? What is the client able to do, and with what kind of intensity? What will the time span be like? And I also ask my client directly that what kind of support does he or she wishes? I'm trying to offer what the client thinks he or she needs" (Finnish nurse 1)

Interestingly, the nurses had different preferences for modes of communication. The Dutch nurses emphasized face-to-face contact and in-person continuity. The Finnish nurses preferred an initial face-to-face consultation but were comfortable with further phone or email contact and did not regard this as less personal than face-to-face contact. Email contact also had advantages:

"But sometimes this kind of communication online could be less complicated…than face to face."(Finnish nurse 5)

"I have noticed in my work that some people prefer contacting me by e-mail and not by phone. [others agree] On the phone they might think that they are disturbing or it's a bad timing, but one can write an e-mail or something anytime." (Finnish nurse 3)

Awareness and expectation management

A second precondition was awareness and expectation management: checking the patients' level of knowledge and expectations regarding prevention and personal cardiovascular risk. Nurses thought that most patients had considerable knowledge of cardiovascular disease prevention, especially in Finland, due to a long standing tradition in community based cardiovascular prevention (the North-Karelia project [26]). Nonetheless, both groups of nurses had the experience that people were not especially aware of their personal cardiovascular risk status:

"That's it, isn't it, for many people their health is not a concern yet. You can list them the facts, and they hear and read it everywhere, that it is unhealthy to have overweight and that they need to exercise more, but right now, they are not yet bothered by it." (Dutch nurse 6)

For peer review only - http://bmjopen.bmj.com/site/about/guidelines.xhtml

Because of this lack of a sense of urgency, the nurses regarded the ability to educate their patients about consequences of health behaviours an essential skill of their profession. Once awareness and motivation had grown, people often had unrealistic expectations and the nurses needed to act as *"myth busters"* (Finnish nurse 4):

> *"And when we are, however, not able to offer the magic pills or wonder tricks, the clients may sometimes be disappointed when all I can suggest is these boring methods: diet and physical activity. And we cannot offer them a magic solution."* (Finnish nurse 4)

Often, once people were motivated to change their health behaviours, they also tended to set unrealistic goals, which the nurses then had to reshape:

> *"Start small. Do not make it too big. If you are obese, many people do not like it to go to the gym, they think the gym is only for lovely slim figures. You cannot convince them that that's not true. Therefore it is important: try things first yourself. What can you do with small steps at home by yourself, before going outside. You have to start liking exercise."* (Dutch nurse 3)

Lastly, the nurses actively prepared their patients for failures during the process of behaviour change, as these were seen as inevitable:

> *"I usually tell the patients that they're allowed to fail; but even so, they are invited to, and they should come to the appointments. So then we can check the situation again, and set a new goal if needed."* (Finnish nurse 1)

With the Finnish nurses, coaching on cardiovascular risk was also related to the potential for dementia prevention. They suggested that many patients feared dementia and lacked knowledge about the disease and treatment and prevention options, creating a stigma towards this condition. The nurses were aware of the link between cardiovascular disease and dementia, but felt they lacked sufficient knowledge and training to provide proper support:

> *"Well, we have not had the knowledge of reasons for dementia for that long. And these connections haven't been…the research is recent: well, at least more recent than the research about heart diseases." (Finnish nurse 5)*

They found that educating patients on the link between cardiovascular disease and dementia, would be a good starting point to raise awareness. Potentially, this could enhance motivation for CV prevention:

For peer review only - http://bmjopen.bmj.com/site/about/guidelines.xhtml

> *"What is good for the heart - and we know what's good for the heart – is also good for the brain but not everyone knows this. I think this link would be good to be aware of: you protect your heart but also the most important part of your body which is the brain."* (Finnish nurse 4)

Appropriate timing and monitoring

The third precondition mentioned by the nurses was appropriate timing and monitoring: providing professional support at appropriate times and monitoring the progress of the patient towards behaviour change. Regular follow-up appointments stimulated adherence and motivation:

> *"After three months, your plan fades away, your goal, your motivation."* (Dutch nurse 3).
>
> *"..that there is a possibility for follow-up. Usually it motivates people when someone looks after you: how are you progressing, no matter if the target is, for example, smoking cessation or increasing of physical activity."* (Finnish nurse 5)

Monitoring ensured that the nurses could support their patients when experiencing obstacles or failures, although this could be difficult:

> *"Disappointments also play a role. For example: a guy with diabetes, he quit smoking but then his sugar levels went up and he needed to start with insulin. How do you explain that [to him]? Well, I challenge you to keep his attitude up and to maintain his motivation."* (Dutch nurse 5)

When discussing monitoring lifestyle behaviours both nurse groups attributed themselves a supportive role putting the patient in charge, because lifestyle was seen as the personal domain of the patient. However, regarding the medical components of preventive care (control of hypertension, diabetes and hypercholesterolemia), the Dutch nurses attributed a more directive role to themselves and the medical practice to avoid mistakes and complications, whereas the Finnish nurses regarded their patients as capable of staying in charge.

Part 2: Integrating the nurses' strategies into an online-support setting

After having identified the preconditions for optimal behaviour change support and the skills nurses use in their current practices, we demonstrated the latest version of the HATICE internet-platform[25] and discussed the online-support setting. Both groups of nurses emphasised the importance of the aforementioned preconditions for optimal online support:

Establishing a relationship of trust

All nurses regarded the presence of a coach as essential to guarantee personal support. The Finnish

For peer review only

nurses felt that online coaching could successfully establish a relationship of trust, provided that the coach was a real person:

> *"Because of this social interaction on this website [the HATICE platform], the participant has a familiar and friendly person [as a coach] and not just some distant virtual coach who is a stranger. [...] it's good that this combines the real-life person with the online contact, maybe it feels more comfortable and familiar [for the participant]."* (Finnish nurse 4)

An initial face-to-face consultation with the patient could strengthen the establishment of a good relationship. Overall, for the Finnish nurses, online support was an obvious step forward in innovating healthcare:

> *"Well at least I think that this is absolutely the trend [others nod and agree], that all the services will be at least partly available online for the patients. Partly like this [via internet] and partly with human contact. I think that it's an inevitable part of future."* (Finnish nurse 1)

In contrast, the Dutch nurses could not imagine the platform and coach fully substituting their personal guidance:

> *"The strength of our guidance is the personal contact we have with the patients. [...] that enables us to give them some subtle support and give them a small push into the right direction. To delegate all of that to an online coach just like that, that seems difficult to me. Then all personal contact will disappear."* (Dutch nurse 7)

Awareness and expectation management

All nurses regarded the internet-platform a suitable mean to raise awareness and increase health-literacy. Managing expectations related to online support was considered very important, because misunderstandings could arise more easily through this mean. Therefore, the coach should explain what could be expected from the platform and their support:

> *"Communication is very important in the beginning:, what is it we do and what do they expect from the goals."* (Dutch nurse 1)

Appropriate timing and monitoring

The nurses envisioned that online, the patient would be in charge of timing of support and monitoring of progress. The coach would have a reactive role, providing support in response to the patient's

For peer review only - http://bmjopen.bmj.com/site/about/guidelines.xhtml

demand. However, the nurses felt the coach also needed to be proactive, in case people showed signs of losing motivation. This would require insight in people's platform activities:

> *"[…] the nurse can also see it [the diary] and check. If the participant fails to achieve the goals, the nurse can go back and check what might have been the problem."* (Finnish nurse 5)

Both groups thought the platform should be aligned to regular healthcare. The Finnish nurses envisioned that the online coach could work in the same fashion as the nurses currently did, targeting both lifestyle and medical components of their patient's health. The Dutch nurses stressed that not everybody would be able to self-manage, especially when it concerned medical issues. Therefore, they preferred a platform focusing on lifestyle only, keeping the control of medical issues in the medical practice:

> *"I think 2 or 3 types of platform users will arise: people who really get the concept of self-management (and start coaching themselves), people who need the coach (and give the coach access to their complete profile) and a group in between, alerting the coach if a goal has not been met."* (Dutch nurse 2)

For peer review only - http://bmjopen.bmj.com/site/about/guidelines.xhtml

DISCUSSION

Principal findings and interpretation

In this international focus group study, we identified three main themes that both the Finnish and Dutch nurses emphasised as most important preconditions for behaviour change support in cardiovascular prevention, and potentially, prevention of cognitive decline: (1) establishing a relationship of trust, (2) managing awareness and expectations and (3) appropriate timing and monitoring of the process of behaviour change. They regarded these preconditions equally important for optimal online support and stressed that a coach providing human support and integration with regular care were essential elements to achieve this. They expressed, however, different ideas on its implementation (**Figure 1**).

Figure 1. Schematic visualization of the main themes and their connections

As mentioned in the introduction, realising and maintaining health behaviour change is notoriously complex. This was confirmed by the nurses we interviewed, but their clinical experience provided us with clear preconditions for optimal behaviour change support. The nurses used slightly different approaches to fulfil these preconditions, both in their current practice and in their ideas on online support. To establish a relationship of trust, the Dutch nurses relied more on face-to-face contact than the Finnish nurses, which appeared to make them more sceptical about the effectiveness of online coaching. The Finnish nurses took a mainly supportive role in monitoring whereas Dutch nurses emphasised a more directive role for themselves and the general practice, with regard to medical aspects of preventive guidance. As **Box 3** shows, the aims of preventive care are very similar between Finland and the Netherlands, with similar roles for primary healthcare nurses. This may explain why the nurses came up with similar preconditions for optimal support of behaviour change. Nevertheless, the differences we found in their current approaches and in their ideas for online support may reflect differences in culture, local healthcare organisation and geography. For example, the nurses' ideas about their own responsibilities and patient autonomy may be aligned to the way patient-autonomy is being shaped in the two healthcare systems as well as to description of nurses' responsibilities in local cardiovascular risk management guidelines. The different attitudes on face-to-face contact can be understood form the perspective of geography. Finland is a large but very sparsely populated country and the Netherlands are a very small but densely populated country. The large distances between patient and health care provider in Finland can make telephone and email contact an attractive alternative for face-to-face consultations. Our results concerning dementia prevention are of special interest. The Finnish nurses liked the idea of including cognitive health as a goal for cardiovascular preventive care, as dementia was regarded a growing public health problem and a combined approach could enlarge people's motivation to engage in behaviour change. However, the nurses felt they could

For peer review only - http://bmjopen.bmj.com/site/about/guidelines.xhtml

not provide proper support, given their limited knowledge and training on one hand, and limited extent of final scientific evidence on the other.

Comparison with existing literature

The experiences of the nurses with behaviour change support were comparable to those described in qualitative studies in other European countries with similar cardiovascular preventive care and as described by Dutch patients [27-31]. The positive attitude of the Finnish nurses on self-management of medical issues was consistent with another Finnish study about nurses' and physicians' perceptions on patient's responsibilities in self-care [32]. The reserved attitude of the Dutch nurses was also reflected in a survey among Dutch healthcare professionals, where 50% feared that patient's direct access to their medical record would cause misunderstandings and unnecessary anxiety[33]. Finally, the conviction of all nurses that a coach was essential to complement the internet-platform, is supported by a meta-analysis we performed showing that internet-interventions combined with human support were more effective than 'stand-alone' interventions [34].

Strengths and limitations

The HATICE project is novel in its aim to develop a generic innovative cardiovascular prevention strategy for older people that can be used across European healthcare systems, especially since it involves eHealth. In qualitative research, international joint analyses are not common because of language barriers. To overcome these, we put much effort in the alignment of our research methodology. The frequent interactions and extensive meetings of the research teams enabled us to explore our findings in the context of the local health care systems. Following grounded theory methodology [24], we deliberately selected nurses that we regarded experts in cardiovascular preventive care. The patient populations of the Finnish and Dutch nurses were not identical with respect to age, which may have influenced our findings. However, since both groups of nurses had comparable experience with cardiovascular prevention and both countries have similar aims for cardiovascular prevention we deem the selection of these nurses appropriate for our research purpose. In addition, the variety in our samples regarding age and clinical experience was large. Since we only performed two focus groups, we cannot exclude that a wider range of views could have been collected For example, one might expect that themes related to training and education requirements would have emerged more prominently from the discussions, but this issue only was mentioned with regard to cognitive health. This issue could be elaborated further in future studies. Further, the striking similarities in the principal themes found in both countries and the consistency of our findings with previous literature mitigates fears that our samples were too limited. Last, when reviewing a summary of our findings, the nurses confirmed that their experiences and views were reflected and did not add new ones, emphasising that the most relevant themes were captured.

For peer review only - http://bmjopen.bmj.com/site/about/guidelines.xhtml

Implications for practice

Finnish and Dutch nurses have similar experiences with and views on supporting behaviour change for cardiovascular prevention, but use different practical approaches towards their patients. Including the maintenance of cognitive health as a goal of cardiovascular prevention might augment people's motivation to partake in health behaviour change. The nurses' experiences provide valuable directions for shaping online support in internet-platforms for cardiovascular self-management. This study also indicates that, when introducing new forms of preventive healthcare that involve patient self-management, like internet-platforms, local healthcare practices are to be taken into account to achieve optimal engagement.

For peer review only

ADDITIONAL INFORMATION

Contributors

Study design: CB, ER, EMvC, HS, FM and MK. Data acquisition: CB, MB and AR. Data analysis: CB, UA, MB and AR. Interpretation of results: CB, UA, MB, AR, FM, EMvC and JP. Drafting of the manuscript: CB. Critical revision of the manuscript for important intellectual content: all authors.

Funding

The research leading to these results has received funding from the European Union Seventh Framework Programme for the HATICE-project (FP7/2007-2013, grant agreement n° 305374) and the Multimodal preventive trials for Alzheimer´s Disease: towards multinational strategies – programme (MIND-AD: MIND-AD Academy of Finland (grant agreement n°291803), MIND-AD VTR, Kuopio University Hospital ((grant agreement n°5772815) and MIND-AD Academy of Sweden (Swedish Research Council, 529-2014-7503)) .

Ethical approval

In the Netherlands, the study was presented to the medical ethics committee of the Academic Medical Centre in the Netherlands and a waiver was provided. In Finland, application for ethical approval nor a waiver were required. Written informed consent was obtained from all participants in both countries.

Competing interests

The authors have declared no competing interests.

Acknowledgements

We thank all Finnish and Dutch nurses for their participation in the study, Suzanne Ligthart, Carin Miedema, Paulien Vermunt, Floor Rooskens (respectively discussion moderator, assistant-moderator and assistants in transcription and coding for the Dutch meetings), Lotta Salo and Ejja Pietilä (organisation and summary notes of the Finnish meeting) for their assistance to the study.

For peer review only - http://bmjopen.bmj.com/site/about/guidelines.xhtml

REFERENCES

1. Oliver D. 21st century health services for an ageing population: 10 challenges for general practice. *The British journal of general practice : the journal of the Royal College of General Practitioners* 2012;62(601):396-7. doi: 10.3399/bjgp12X653435 [published Online First: 2012/08/08]
2. Wu YT, Fratiglioni L, Matthews FE, et al. Dementia in western Europe: epidemiological evidence and implications for policy making. *The Lancet Neurology* 2016;15(1):116-24. doi: 10.1016/s1474-4422(15)00092-7 [published Online First: 2015/08/25]
3. de Bruijn RF, Bos MJ, Portegies ML, et al. The potential for prevention of dementia across two decades: the prospective, population-based Rotterdam Study. *BMC medicine* 2015;13:132. doi: 10.1186/s12916-015-0377-5 [published Online First: 2015/07/22]
4. Kivipelto M, Ngandu T, Laatikainen T, et al. Risk score for the prediction of dementia risk in 20 years among middle aged people: a longitudinal, population-based study. *The Lancet Neurology* 2006;5(9):735-41. doi: 10.1016/s1474-4422(06)70537-3 [published Online First: 2006/08/18]
5. Norton S, Matthews FE, Barnes DE, et al. Potential for primary prevention of Alzheimer's disease: an analysis of population-based data. *The Lancet Neurology* 2014;13(8):788-94. doi: 10.1016/s1474-4422(14)70136-x [published Online First: 2014/07/18]
6. Peters R, Beckett N, Forette F, et al. Incident dementia and blood pressure lowering in the Hypertension in the Very Elderly Trial cognitive function assessment (HYVET-COG): a double-blind, placebo controlled trial. *The Lancet Neurology* 2008;7(8):683-9. doi: 10.1016/s1474-4422(08)70143-1 [published Online First: 2008/07/11]
7. Leventhal H, Weinman J, Leventhal EA, et al. Health psychology: the search for pathways between behavior and health. *Annu Rev Psychol* 2008;59:477-505.
8. Rosenstock IM. The Health Belief Model and Preventive Health Behavior. *Health Education Monographs* 1974;2(4):354-86. doi: 10.1177/109019817400200405
9. Ajzen I. From Intentions to Actions: A Theory of Planned Behavior. In: Kuhl J, Beckmann J, eds. Action Control: From Cognition to Behavior. Berlin, Heidelberg: Springer Berlin Heidelberg 1985:11-39.
10. Bandura A. Self-efficacy: Toward a unifying theory of behavioral change. *Psychological Review* 1977;84(2):191-215. doi: 10.1037/0033-295X.84.2.191
11. Prochaska JO, DiClemente CC, Norcross JC. In search of how people change. Applications to addictive behaviors. *The American psychologist* 1992;47(9):1102-14. [published Online First: 1992/09/01]
12. Weinstein ND. The precaution adoption process. *Health Psychology* 1988;7(4):355-86. doi: 10.1037/0278-6133.7.4.355
13. Burke LE, Dunbar-Jacob JM, Hill MN. Compliance with cardiovascular disease prevention strategies: a review of the research. *Ann Behav Med* 1997;19(3):239-63.
14. Abraham C, Kelly MP, West R, et al. The UK national institute for health and clinical excellence public health guidance on behaviour change: A brief introduction. *Psychology, Health & Medicine* 2009;14(1):1-8. doi: 10.1080/13548500802537903
15. Busse R, Blümel M, Scheller-Kreinsen D, et al. Tackling chronic disease in Europe Strategies, interventions and challenges. *World Health Organization 2010, on behalf of the European Observatory on Health Systems and Policies* 2010;20
16. Bodenheimer TL, K.; Holman, H.; Grumbach, K. Patient Self-Management of Chronic Disease in Primary Care. *Jama* 2002;288(19):2469 - 75.
17. Lorig K, Holman H. Self-Management Education: History, Definition, Outcomes, and Mechanisms. *Ann Behav Med* 2003;26(1):1-7.
18. Griffiths F, Lindenmeyer A, Powell J, et al. Why are health care interventions delivered over the internet? A systematic review of the published literature. *Journal of medical Internet research* 2006;8(2):e10. doi: 10.2196/jmir.8.2.e10 [published Online First: 2006/07/27]
19. Bennett GG, Glasgow RE. The delivery of public health interventions via the Internet: actualizing their potential. *Annual review of public health* 2009;30:273-92. doi: 10.1146/annurev.publhealth.031308.100235 [published Online First: 2009/03/20]

For peer review only - http://bmjopen.bmj.com/site/about/guidelines.xhtml

20. Richard E, Jongstra S, Soininen H, et al. Healthy Ageing Through Internet Counselling in the Elderly: the HATICE randomised controlled trial for the prevention of cardiovascular disease and cognitive impairment. *BMJ Open* 2016;6(6):e010806. doi: 10.1136/bmjopen-2015-010806 [published Online First: 2016/06/12]
21. Glaser BG, Strauss AL. The discovery of grounded theory. *Aldine, Chicago* 1967
22. Strauss A, Corbin J. Grounded theory methodology. *Handbook of qualitative research* 1994:273-85.
23. Tong A, Sainsbury P, Craig J. Consolidated criteria for reporting qualitative research (COREQ): a 32-item checklist for interviews and focus groups. *International Journal for Quality in Health Care* 2007;19(6):349-57. doi: 10.1093/intqhc/mzm042
24. Foley G, Timonen V. Using Grounded Theory Method to Capture and Analyze Health Care Experiences. *Health Services Research* 2015;50(4):1195-210. doi: 10.1111/1475-6773.12275
25. Jongstra S, Beishuizen C, Andrieu S, et al. Development and Validation of an Interactive Internet Platform for Older People: The Healthy Ageing Through Internet Counselling in the Elderly Study. *Telemedicine journal and e-health : the official journal of the American Telemedicine Association* 2016 doi: 10.1089/tmj.2016.0066 [published Online First: 2016/07/28]
26. Puska P, Nissinen A, Tuomilehto J, et al. The community-based strategy to prevent coronary heart disease: conclusions from the ten years of the North Karelia project. *Annual review of public health* 1985;6:147-93. doi: 10.1146/annurev.pu.06.050185.001051 [published Online First: 1985/01/01]
27. Loon MS, van Dijk-de Vries A, van der Weijden T, et al. Ethical issues in cardiovascular risk management: Patients need nurses' support. *Nursing ethics* 2014;21(5):540-53. doi: 10.1177/0969733013505313 [published Online First: 2013/11/22]
28. Ligthart SA, van den Eerenbeemt KD, Pols J, et al. Perspectives of older people engaging in nurse-led cardiovascular prevention programmes: a qualitative study in primary care in the Netherlands. *The British journal of general practice : the journal of the Royal College of General Practitioners* 2015;65(630):e41-8. doi: 10.3399/bjgp15X683149 [published Online First: 2014/12/31]
29. Brobeck E, Bergh H, Odencrants S, et al. Primary healthcare nurses' experiences with motivational interviewing in health promotion practice. *Journal of clinical nursing* 2011;20(23-24):3322-30. doi: 10.1111/j.1365-2702.2011.03874.x [published Online First: 2011/10/25]
30. Boase S, Mason D, Sutton S, et al. Tinkering and tailoring individual consultations: how practice nurses try to make cardiovascular risk communication meaningful. *Journal of clinical nursing* 2012;21(17-18):2590-8. doi: 10.1111/j.1365-2702.2012.04167.x [published Online First: 2012/07/14]
31. Hornsten A, Lindahl K, Persson K, et al. Strategies in health-promoting dialogues--primary healthcare nurses' perspectives--a qualitative study. *Scandinavian journal of caring sciences* 2014;28(2):235-44. doi: 10.1111/scs.12045 [published Online First: 2013/04/19]
32. Jallinoja P, Absetz P, Kuronen R, et al. The dilemma of patient responsibility for lifestyle change: Perceptions among primary care physicians and nurses. *Scand J Prim Health Care* 2007;25(4):244-49. doi: 10.1080/02813430701691778
33. Krijgsman J, Peeters J, Burghouts A, et al. Between the spark and the flame: 2015 eHealth monitor. *Nictiz and the NIVEL Institute [Dutch Institute for Research in the Healthcare Sector]* 2015;October
34. Beishuizen CR, Stephan BC, van Gool WA, et al. Web-Based Interventions Targeting Cardiovascular Risk Factors in Middle-Aged and Older People: A Systematic Review and Meta-Analysis. *Journal of medical Internet research* 2016;18(3):e55. doi: 10.2196/jmir.5218 [published Online First: 2016/03/13]
35. Hämäläinen P, Reponen J, Winblad I. eHealth of Finland - Checkpoint 2008. 2009
36. Säkkinen S, Heino A, Häkkinen P, et al. Statistical yearbook on social welfare and health care 2015 - National Institute for Health and Welfare. 2016
37. Vuorenkoski L, Mladovsky P, Mossialos E. Finland: Health system review. *Health Systems in Transition* 2008;10(4):1-168.
38. http://www.kanta.fi/en [Website of the Finnish Patient Data Repository, where Finnish people can access their own medical record]. accessed 7-12-2016 2016.

For peer review only - http://bmjopen.bmj.com/site/about/guidelines.xhtml

39. http://www.kaypahoito.fi/web/english/guidelineabstracts/guideline?id=ccs00014 [Finnish Current Care Guideline on hypertension (2014)]. accessed 7-12-2016 2016.
40. http://www.kaypahoito.fi/web/english/guidelineabstracts/guideline?id=ccs00032 [Finnish Current Care Guideline on diabetes (2016)]. accessed 7-12-2016 2016.
41. http://www.kaypahoito.fi/web/english/guidelineabstracts/guideline?id=ccs00055 [Finnish Current Care Guideline on dyslilidaemias (2013)]. accessed 7-12-2016 2016.
42. Ministry of Public Health, Welfare and Sport - Healthcare in the Netherlands. 2016
43. Schäfer W, Kroneman M, Boerma W, et al. The Netherlands: Health system review *Health Systems in Transition* 2010;12(1):1-229.
44. Boomsma L, Lakerveld-Heyl K, Gorter K, et al. Landelijke Eerstelijns Samenwerkings Afspraak Diabetes mellitus type 2. *Huisarts Wet* 2006;49(8):418-24.
45. den Boer J. Protocollair CVRM: Nederlands Huisartsen Genootschap 2016.
46. Faber E, De Bont M, Beusmans G, et al. Landelijke Eerstelijns Samenwerkings Afspraak CVA. *Huisarts Wet* 2007;50(3):S5-S8.
47. Kringos D, Boerma W, Bourgueil Y, et al. The strength of primary care in Europe: an international comparative study. *The British journal of general practice : the journal of the Royal College of General Practitioners* 2013;63(616):e742-e50. doi: 10.3399/bjgp13X674422

For peer review only

For peer review only - http://bmjopen.bmj.com/site/about/guidelines.xhtml

BOXES

Box 1. Main topics discussed

**Part 1**
• Prevention of cardiovascular disease and dementia: attitude and experiences
• Good guidance of behaviour change
• Relationship with the patient
**Part 2**
• Attitude towards the internet-platform and online-support
• Role and responsibilities of the internet-coach
• Interaction with the patients online

Box 2. Key features of the HATICE internet-platform with coaching

• Patient-centred: the patient can login onto a personal portal to review and manage his/her personal cardiovascular risk profile
• Improving health knowledge: the patient can access educational modules about cardiovascular risk factors and lifestyle
• Goal setting and self-monitoring: the patient can set his/her own goals for behaviour change and monitor how he/she is doing by entering self-measurements or keeping a diary
• Coaching: the coach monitors the patient's self-management and they can communicate online through messages

For peer review only - http://bmjopen.bmj.com/site/about/guidelines.xhtml

Box 3: the Finnish and Dutch primary care systems

Both Finland and the Netherlands have strongly developed primary care systems with an important gatekeeper function:

The Finnish primary care system

In Finland, health promotion and disease prevention have been the focus of healthcare policy for decades. Primary care is delivered by public healthcare centres but also through occupational health facilities[35-37]. In many parts of Finland, healthcare centres cover large geographical areas that are sparsely populated and often have shortage of staff, contributing to long waiting lists and lack of personal continuity of care. All healthcare centres use electronic medical records to ensure continuity of care. Finland was the first European country to introduce a law (in 1993) defining the patient's right to access to all medical information and the right to autonomy (patient's informed consent for any medical treatment). Currently, a national patient data repository is under development to provide Finnish patients complete access to their own electronic medical record[38]. Nurses have an important role in primary healthcare. They work in close collaboration with the general practitioners and have their own consulting hours to assess patients. Regarding cardiovascular prevention, they monitor patients with diabetes, hypertension and dyslipidaemia, as described in national guidelines[39-41]. Finnish companies offer occupational health facilities to their employees, including both preventive and curative health services, which are delivered through semi-private healthcare centres that also work with nurses in a similar fashion as the public primary health care centres. Since waiting lists are long in public primary care, many employees direct themselves to these health services instead.

The Dutch primary care system

Key features of the Dutch healthcare system are access to care for everyone and solidarity through medical insurance[42 43]. General practices form the core of primary care and general practitioners (GPs) are gatekeepers of the healthcare system, providing acute, chronic and preventive care. Since the Netherlands are densely populated, people often live at short distance from their general practice. In most general practices continuity of care is ensured by allocating the patient to one GP. In the Netherlands, informed consent is also ensured by law, but in daily practice, consent is often assumed and only explicitly discussed in case treatment options can have far-reaching consequences[43]. Almost all GPs use electronic medical records. Patients have the right to inspect their medical records, but do not have complete access to them.

Nurses have an important position in primary care in the Netherlands. Since several decades, GPs have delegated tasks to practice nurses, especially concerning chronic disease management. Currently, these nurses provide a substantial part of cardiovascular risk management care, including diabetes care, which has been worked out in several regional and national guidelines and work descriptions[9 44-46]. Access to the GP is efficient, there are no waiting lists[47].

For peer review only - http://bmjopen.bmj.com/site/about/guidelines.xhtml

FIGURE LEGENDS

Figure 1. Schematic visualization of the main themes and their connections

Legend: Left, the three main preconditions for good behaviour change guidance in cardiovascular preventive care that both Finnish and Dutch nurses identified, are depicted. Right of this, it is shown how the Finnish and Dutch nurses suggest to realise these preconditions in the online setting. Since there were differences between the nurses this is depicted separately for the Finnish and Dutch nurses. Below it is shown that local health practices influenced both the preconditions (and their operationalization (not shown in figure but explained in results section)) and the integration into online support.

For peer review only

For peer review only - http://bmjopen.bmj.com/site/about/guidelines.xhtml

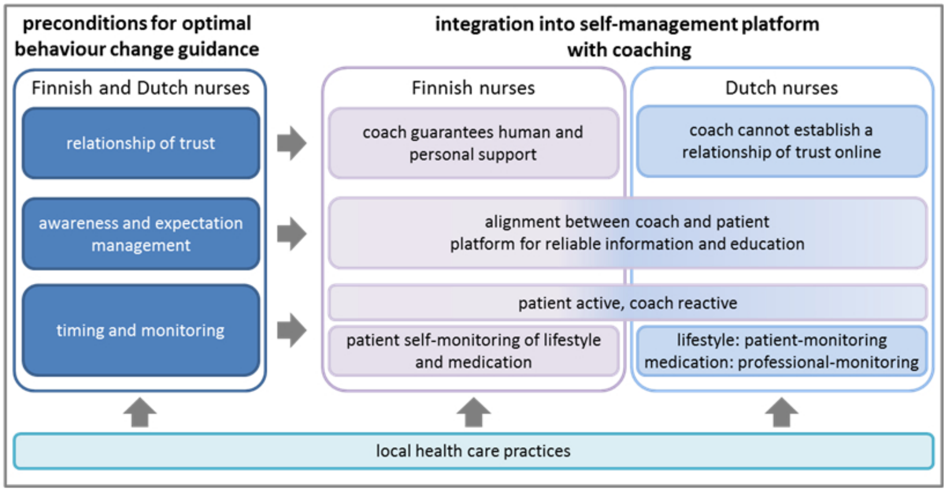

Caption : Caption: Figure 1. Schematic visualization of the main themes and their connectionsLegend: Left, the three main preconditions for good behaviour change guidance in cardiovascular preventive care that both Finnish and Dutch nurses identified, are depicted. Right of this, it is shown how the Finnish and Dutch nurses suggest to realise these preconditions in the online setting. Since there were differences between the nurses this is depicted separately for the Finnish and Dutch nurses. Below it is shown that local health practices influenced both the preconditions (and their operationalization (not shown in figure but explained in results section)) and the integration into online support.

228x121mm (96 x 96 DPI)

APPENDIX 1 to 'Integrating nurses' experiences with supporting behaviour change for cardiovascular prevention into a self-management internet-platform in Finland and the Netherlands: a qualitative study'

Appendix 1: COREQ checklist

No.	Item	Explanation	Reported on page no.
Domain 1:Research team and reflexivity			
1	Interviewers	NL: Pols AJ and Ligthart S<br>FI: Rosenberg A	Not reported in manuscript
2	Credentials of the researchers involved in data collection and analysis	NL: Beishuizen CRL: MD PhD-student; Rooskens F: BSc; Lighthart S: MD, PhD-student; Pols AJ: prof; Moll van Charante EP: MD PhD<br>FI: Akenine U: PhD-student; Barbera M: PhD; Rosenberg A: PhD-student	Not reported in manuscript
3	Occupation of the researchers involved in data collection and analysis	NL: Beishuizen CRL: PhD-student; Rooskens F: medical student; Ligthart S: general practitioner in training, PhD-student; Pols AJ: professor in medical ethics; Moll van Charante EP: postdoc researcher, general practitioner<br>FI: Akenine U: PhD-student, research nurse; Barbera M: postdoc researcher; Rosenberg A: PhD-student	Not reported in manuscript
4	Gender	All researchers are female, except for Moll van Charante EP, who is male	Not reported in manuscript
5	Experience and training	See main text	p5
6	Relationship established	NL: there was an indirect relationship between research team and the partcipants, because the nurses had been involved in a previous research project of the research team in which 2 researchers (Ligthart S and Moll van Charante EP) were also involved<br>FI: no previous relationship established	Not reported in manuscript
7	Partcipant knowledge of the	NL: participants knew the professional background of the moderators	Not reported in

For peer review only - http://bmjopen.bmj.com/site/about/guidelines.xhtml

	interviewer	FI: participants knew the professional background of the moderator	manuscript
8	Interviewer characteristics	NL: Pols AJ conducts qualitative research on the ethics of use of technology and eHealth in medical care. Ligthart S conducts quantitative and qualitative research on cardiovascular prevention in older people<br><br>FI: Rosenberg A conducts research on prevention of dementia	Not reported in manuscript
Domain 2: Study design			
9	Methodological information and theory	See main text	p4 and p5
10	Sampling	See main text	p4
11	Method of approach	See main text	p4
12	Sample size	See main text	p4
13	Non-participation	See main text	p4
14	Setting of data collection	See main text	p4
15	Presence of non-participants	NL: Eric Moll- van Charante and Pim Happel were present as non-participating audience<br><br>FI: no others were present	Not reported in manuscript
16	Description of sample	See main text	p4
17	Interview guide	See main text	p5
18	Repeat interviews	Not performed	
19	Audio/visual recording	See main text	p5
20	Field notes	See main text	p5
21	Duration	See main text	p5

For peer review only - http://bmjopen.bmj.com/site/about/guidelines.xhtml

22	Data saturation	See main text	P1
23	Transcripts returned	Not performed	
Domain 3: analysis and findings			
24	Number of data coders	NL: 2<br>FI: 2	p5
25	Description of the coding tree	See main text (figure 1)	p11
26	Derivation of themes	See main text	p5
27	Software	No special qualitative software was used	
28	Participant checking	See main text	p6 and p13
29	Quotations presented	See main text	p6 – p11
30	Data and findings consistent	See main text	p6 – p11
31	Clarity of major themes	See main text and figure 1	p6-p12
32	Clarity of minor themes	Within the groups, the Dutch and Finnish nurses shared opinions and experiences on most topics. Between the Dutch and Finnish groups, some interesting differences in opinions and experiences were identified. We choose therefore to focus on these differences when presenting our results, but not on diverse cases within the groups.<br><br>Minor themes were not discussed due to word limits	Not reported in manuscript

For peer review only

APPENDIX 2 to 'Integrating nurses' experiences with supporting behaviour change for cardiovascular prevention into a self-management internet-platform in Finland and the Netherlands: a qualitative study'

Appendix 2: Topic list

**FOCUS GROUP nurses part ONE cardiovascular prevention**		
**Overview part one**	**Topic (possible items)**	**Possible questions**
Aim of the meeting is to learn from the nurses' experiences in cardiovascular prevention: what are best practices to stimulate behaviour change, what is their attitude towards a prevention program for older people via the internet, how would they support people in lifestyle change through the internet?		
	**CVD prevention**	
	Activities on CVD prevention	Do you currently conduct activities on CVD prevention?
	Experiences / lessons learned	What are your experiences and lessons learned, especially regarding giving 'medical' guidance versus giving lifestyle guidance and regarding behavior change?
	**Dementia prevention**	In the last decade, we got more and more indications from neurological research that risk factors for cardiovascular disease are also risk factors for dementia. So, possibly, reducing cardiovascular risk may also postpone or prevent dementia.
	Attitude towards dementia prevention	What is your attitude / your ideas regarding dementia prevention?
	Awareness of dementia risk and compliance to lifestyle change	Most people are not yet aware of the association between CV risk and dementia, but do seem to be very afraid of dementia. Do you think that more awareness would enhance compliance/adherence to lifestyle change? What are your ideas on this?
	**Relationship with participants and regular healthcare system**	
	Guiding lifestyle change	Which factors could contribute to good guidance of lifestyle change?
	Relationship with patient	Which factors could contribute to a good relationship with your patient?
	Relation with GP and regular practice assistant	How should the platform coach link with the patients' GP and regular practice assistant/nurse (the regular healthcare system)?

For peer review only - http://bmjopen.bmj.com/site/about/guidelines.xhtml

	Attitude towards internet intervention	What is your attitude towards a prevention program via the internet (with support from a coach)?
		How should the platform coach link with the patients' GP and regular practice assistant/nurse (the regular healthcare system)?
**FOCUS GROUP nurses part TWO The platform**		
Assistant moderator explains about HATICE and platform by showing the powerpoint with screen shots of the platform		
	**Information required for support**	Imagine yourself being a coach using the internet-platform:
		Which kind of information regarding the participants do you need to be able to support them?
	**Role and responsibilities**	
	Responsibility goal setting	Who is responsible for goal setting? (capability of patients)
	Role in lifestyle groups	How do you see your role in creating lifestyle groups and how can participation be encouraged?
	**Interaction with participants**	
	Experience with motivational interviewing	Do you have experience with motivational interviewing, how could this technique be used by the coach?
	Frequency of contact	How often would you like to have contact with your patient?
	Mode of communication	Which mode of communication do you prefer? (phone, skype +/- webcam, email, chat…)
	Network of support	What should be the role of the participant network of support in lifestyle change and how can this network be engaged?
	Alerts / reminders	Do you like to receive automatic alerts/reminders when patients have alarming values or did not log-on?
Assistant moderator and moderator verify key messages from focus group		
Moderator thanks nurses		

For peer review only - http://bmjopen.bmj.com/site/about/guidelines.xhtml

BMJ Open

Integrating nurses’ experiences with supporting behaviour change for cardiovascular prevention into a self-management internet-platform in Finland and the Netherlands: a qualitative study

Journal:	*BMJ Open*
Manuscript ID	bmjopen-2018-023480.R1
Article Type:	Research
Date Submitted by the Author:	20-Dec-2018
Complete List of Authors:	Beishuizen, Cathrien; Academic Medical Center, General Practice<br>Akenine, Ulrika; Karolinska Institutet and Karolinska University Hospital, Division of Clinical Geriatrics , Department of Neurobiology, Care Sciences and Society<br>Barbera, Mariagnese; University of Eastern Finland, Clinical Medicine/Neurology<br>Rosenberg, Anna; University of Eastern Finland, Clinical Medicine/Neurology<br>Fallah Pour, Mandana; Karolinska Institutet, Division of Occupational Therapy, Department of Neurobiology, Care Sciences and Society<br>Richard, Edo; Academic Medical Centre, University of Amsterdam, Neurology; Radboud University Medical Center, Department of Neurology, Donders Institute for Brain, Cognition and Behaviour<br>Soininen, Hilkka; University of Eastern Finland, Clinical Medicine/Neurology<br>Mangialasche, Francesca; Karolinska Institutet, Division of Geriatric Epidemiology<br>Kivipelto, Miia; Karolinska Institutet, Division of Geriatric Epidemiology; University of Eastern Finland, Clinical Medicine/Neurology<br>Pols, A.; AMC, Department of General Practice, Section of Medical Ethics,<br>Moll van Charante, Eric; Academic Medical Center, General Practice
**Primary Subject Heading**:	Cardiovascular medicine
Secondary Subject Heading:	General practice / Family practice, Health services research, Nursing, Patient-centred medicine, Qualitative research
Keywords:	QUALITATIVE RESEARCH, PRIMARY CARE, Nurse-led care, eHealth, Cardiovascular prevention, Dementia prevention

SCHOLARONE™ Manuscripts

Integrating nurses' experiences with supporting behaviour change for cardiovascular prevention into a self-management internet-platform in Finland and the Netherlands: a qualitative study

Authors

Beishuizen CRL[1*], Akenine U[2], Barbera M[3], Rosenberg A[3], Fallahpour M[4], Richard E[5,6], Soininen H[3,7], Mangialasche F[8], Kivipelto M[2, 3,9], Pols AJ[10] and Moll van Charante EP[1]

*corresponding author

address: Department of General Practice
Amsterdam UMC/University of Amsterdam
Meibergdreef 9, 1100 DD
Amsterdam, Netherlands

email: c.r.beishuizen@amc.uva.nl

phone: +31 6 16359453

Affiliations

[1] Department of General Practice, Amsterdam UMC/University of Amsterdam, Amsterdam, The Netherlands
[2] Division of Clinical Geriatrics, Department of Neurobiology, Care Sciences and Society, Karolinska Institutet, and Karolinska University Hospital, Stockholm, Sweden
[3] Institute of Clinical Medicine/Neurology, University of Eastern Finland, Kuopio, Finland
[4] Department of Neurobiology Care Sciences and Society, Division of Occupational Therapy, Karolinska Institutet, Huddinge, Sweden
[5] Department of Neurology, Amsterdam UMC/University of Amsterdam, Amsterdam, The Netherlands
[6] Department of Neurology, Donders Institute for Brain, Cognition and Behaviour, Radboud University Medical Center, Nijmegen, The Netherlands
[7] Neurocenter, Neurology, Kuopio University Hospital, Kuopio, Finland.
[8] Aging Research Center, Karolinska Institutet and Stockholm University, Stockholm, Sweden
[9] Chronic Disease Prevention Unit, National Institute for Health and Welfare, Helsinki,, Finland
[10] Section of Medical Ethics, Department of General Practice, Amsterdam UMC/University of Amsterdam, Amsterdam, The Netherlands

Word count

Abstract: 258

Total text (main text and quotations): 4,838

Main text only: 3,738

Quotations only: 1,100

For peer review only - http://bmjopen.bmj.com/site/about/guidelines.xhtml

Abstract

Objectives: Global ageing is linked to an increased burden of cardiovascular disease (CVD) and dementia, which calls for better prevention strategies. Self-management and eHealth applications are regarded as promising strategies to support prevention. The aim of this study was to explore nurses' best practices with behaviour change guidance for cardiovascular (CV) prevention in order to learn how to optimally integrate these into a coach-supported internet-platform for CV self-management.

Design: Qualitative focus group study in Finland and the Netherlands. Discussions were audiotaped and transcribed. Data were thematically analysed following grounded theory.

Setting: Dutch and Finnish primary care settings.

Participants: Six Finnish and seven Dutch primary care nurses experienced in CV prevention

Results: Similar best practices were found and comprised of: (1) establishing a relationship of trust, (2) managing awareness and expectations and (3) appropriate timing and monitoring of the process of behaviour change. However, the Finnish and Dutch nurses used different approaches for accomplishment of these practices, which was reflected in their recommendations for online-support. Both groups emphasised that online-support should be combined with human-support and integrated in regular care. Finnish nurses had more confidence in patient self-management and remote communication than Dutch nurses, who emphasised the importance of face-to-face contact and preferred to keep the control on medical aspects of prevention.

Conclusions: Differences in CV prevention support of Dutch and Finnish nurses appear to reflect their local healthcare practices, which should be taken into account when designing internet-platforms for health self-management. Including cognitive health as a goal of CV prevention might stimulate people's motivation for health behaviour change.

Keywords: cardiovascular prevention, dementia prevention, behaviour change, eHealth, primary care, nurse-led care, qualitative research

Strengths and limitations of this study

- This international focus group study directly compares best practices of Finnish and Dutch primary care nurses in cardiovascular prevention
- Language barriers were overcome by closely aligning our research methodologies, multiple iterations in the analysis and extensive meetings between the research teams
- Our original approach, following grounded theory, enabled us to learn from the best practices of nurse experts in 'traditional' face-to-face cardiovascular preventive care and integrate these into optimal health behaviour change support through novel eHealth applications

For peer review only - http://bmjopen.bmj.com/site/about/guidelines.xhtml

- Since our samples and number of focus groups were limited, we are aware that our findings are not exhaustive, especially since we identified local health care practices substantially impacted the study results
- Due to the exploratory character of our study, our findings are preliminary and might be influenced by differences in clinical experience of the nurses and differences in age of their patient populations

For peer review only

For peer review only - http://bmjopen.bmj.com/site/about/guidelines.xhtml

Manuscript

INTRODUCTION

Global ageing places an increasing demand on healthcare systems, partially due to the absolute rise in cardiovascular disease (CVD) and dementia cases [1 2]. As these disorders share a number of risk factors, effective cardiovascular (CV) prevention could also lead to the prevention of dementia [3-6]. CV prevention requires health behaviour change, the process of "initiating and maintaining behaviours that reduce health risks and control existent chronic disease"[7]. In CV prevention, core behaviours consist of a healthy lifestyle (healthy diet, sufficient physical activity and non-smoking) and adherence to medication. Although the processes behind supporting health behaviour change have been theorised extensively[8-12], putting them into practice remains a challenge[13 14] and novel, more effective, approaches are needed[15]. Two strategies of current interest are self-management and eHealth. In self-management, the individual, instead of the healthcare professional, takes the lead in the management of his/her risk factors and adherence, and therefore in behaviour change[16 17]. eHealth applications are attractive because of their wide reach and have the potential to support self-management because of their suitability for health education, interactivity and monitoring[18 19]. Although researchers and policymakers have high expectations of eHealth and self-management, the more intensive face-to-face interventions still achieve better results than eHealth applications[20]. To learn how self-management and behaviour change are best stimulated and maintained online, we consulted 'experts in the field': nurses experienced in health behaviour change in the context of CV prevention.

This project is part of the Healthy Ageing Through Internet Counselling in the Elderly (HATICE) study, which includes a European randomised controlled trial testing a coach-supported internet-platform for self-management of cardiovascular risk factors in older people to prevent CVD and cognitive decline [21]. In an international focus group study, we aimed to explore (1) nurses' best practices with behaviour change guidance for cardiovascular prevention, including the potential for dementia prevention, and (2) learn how to integrate their practices into a coach-supported internet-platform (the online-support setting). This study took place in Finland and the Netherlands, two of the three countries that participated in the HATICE-study. Since the HATICE project aims to develop an internet-platform that is implementable across all European healthcare systems, we also explored the influence of local healthcare practices.

For peer review only - http://bmjopen.bmj.com/site/about/guidelines.xhtml

METHODS

Design

We performed an international qualitative focus group study following grounded theory[22 23]. The COREQ-checklist is included for complete information on methodology (**Appendix 1**)[24].

Participants and setting

For sampling, we followed the grounded theory methodology of studying a health care practice by consulting field experts [25]. In this light, Finnish and Dutch primary care nurses experienced in CV preventive care were most eligible for this study and selective purposive samples were obtained. In Finland, occupational healthcare nurses were recruited because of their important role in preventive CV care (please see **Box 1** for a description of Finnish and Dutch primary health care systems and the position of Finnish occupational health). Fourteen nurses working in a semi-private healthcare centre in Kuopio (Eastern Finland) were invited by email and telephone and six female nurses (43%) consented to participate. Being occupational health nurses they cared mostly for patients in the working age. Clinical experience with CV prevention ranged from 2 to 35 years. In the Netherlands, we recruited primary care nurses experienced in cardiovascular risk management. A group of 32 nurses experienced in CV preventive care working in general practices in two urban areas in the centre of the Netherlands was invited by email and telephone. Seven female nurses (22%) consented to participate. The unanimous reason for non-participation by Finnish and Dutch nurses was lack of time. The Dutch participating nurses cared for patients of all ages. Clinical experience with CV prevention ranged from 3 to 11 years. **Table 1** contains further characteristics.

Box 1. The Finnish and Dutch primary care systems

Table 1. Characteristics of the participating Finnish and Dutch nurses

Nr	Country*	Age	Education	Typ of CVD prevention	Internet use at work
**1**	FI	55	occupational health nurse	prim/sec prev	email, guideline use, referral, patient contact
**2**	FI	42	occupational health nurse	prim/sec prev	email, guideline use, referral
**3**	FI	25	occupational health nurse	prim/sec prev	email, guideline use, referral, patient contact
**4**	FI	45	occupational health nurse	prim/sec prev	email, guideline use, referral, patient contact
**5**	FI	49	occupational health nurse	prim/sec prev	email, guideline use, referral, patient contact
**6**	FI	60	occupational health nurse	prim/sec prev	guideline use, patient contact
**1**	NL	43	general nurse, practice nurse[a]	prim/sec prev	email, guideline use, referral, patient contact

For peer review only - http://bmjopen.bmj.com/site/about/guidelines.xhtml

**2**	NL	49	practice nurse	prim/sec prev	email, guideline use, referral, patient contact
**3**	NL	51	practice nurse	prim prev	email, guideline use, referral, patient contact
**4**	NL	53	general nurse, practice nurse	prim/sec prev	email, guideline use, referral
**5**	NL	42	practice nurse	sec prev	email, guideline use, referral, patient contact
**6**	NL	45	general nurse, practice nurse[b]	prim/sec prev	email, guideline use, referral, patient contact
**7**	NL	65	general nurse, practice nurse	prim/sec prev	email, guideline use, referral, patient contact

*abbreviations: CVD = cardiovascular disease, FI = Finland, prim = primary, sec = secondary, prev = prevention, NL = the Netherlands, DM = diabetes mellitus, COPD = chronic obstructive pulmonary disease

[a] practice nurse: received specific nursing training to work in the general practice
[b] general nurse: received general nursing training to work as a general nurse in the hospital

The study was presented to the medical ethics committee of the Academic Medical Centre in the Netherlands and a waiver was provided. In Finland, application for ethical approval nor a waiver were required. All participants provided written informed consent.

Data collection

We conducted one focus group in the Netherlands (autumn 2013) and one in Finland (December 2015). We regarded focus groups the most appropriate method to answer our research aims, because it enabled us to explore the experiences and attitudes of the nurses most completely, as the nurses could directly respond to each other's opinions and develop their ideas through the discussion. In each country, an experienced focus group moderator chaired the sessions, while an assistant-moderator noted non-verbal communication and summarised the discussions. The discussion was conducted using a topic list as a flexible guide (**Box 2** and **Appendix 2**). After the Dutch session, the topic list was refined for the Finnish focus group, to further explore the following topics: the nurse-patient relationship, attitude towards eHealth applications, shaping optimal online support, the role of the nurse in this versus the role of the patient and dementia prevention. The moderators asked open-ended questions, following grounded theory to inductively approach the data. Both moderators first asked the nurses about their activities in cardiovascular prevention and how they supported their patients in the process of behaviour change. The Finnish moderator also asked the nurses about their experiences on prevention of dementia. In the second part, the HATICE internet-platform was presented (**Box 3**, a full description of the platform is reported elsewhere [26]) and the nurses were asked how they would optimally support their patients in an online setting. Both sessions lasted approximately two hours. The discussions were audio-recorded and transcribed.

Box 2. Main topics discussed

For peer review only - http://bmjopen.bmj.com/site/about/guidelines.xhtml

Box 3. Key features of the HATICE internet-platform with coaching

Coding and analysis

In each country, two researchers coded and thematically analysed the transcripts following grounded theory [22 23]. Themes were derived inductively from the data and were not hypothesised beforehand. Open coding and identification of initial themes was first performed by the two researchers independently. Thereafter, codes and themes were compared. Dissimilarities were discussed until consensus was reached. Initial theme structure was then discussed with the senior researchers involved. In Finland, since the researchers were not Finnish native speakers, the transcript was translated into English and cross-checked by the Finnish focus group moderator, who was a Finnish native fluent English speaker. In this way, the complete analysis of the Finnish data could be performed in English. After the initial analysis performed locally, themes and corresponding quotations of the Dutch sessions were also translated into English. The two research teams then had two meetings to discuss the structure of main themes and categories. The analysis-phase[22 23] was an iterative process, during which the researchers of both teams repeatedly returned to their data-files to add, merge and refine themes, until a definite theme structure was agreed on by all authors. During the iterative analysis-phase, the researchers discussed the themes and alternatives and it was proposed that the local health care context was of influence on the differences found between caring styles of the two groups of nurses. Therefore, the research teams introduced their local health care systems (**Box 3**) to each other and these insights were used in further interpretation of the findings. A summary of the final conclusions was returned to the participants for feedback.

Patient and public involvement

Patients were not involved in the design of this substudy of HATICE. However, patients were involved in the development of the HATICE eHealth application by means of conduction focus groups with the projected target population of the HATICE eHealth application and by means of consulting patient organisations (Dutch Heart Foundation and Dutch and Finnish Alzheimer Association)[26]. Results of this substudy were disseminated to the participants by means of a written summary.

RESULTS

We present our findings in two sections: 1) Nurses experiences and practices with supporting the process of behaviour change for cardiovascular prevention, including the potential for dementia prevention, and 2) Their suggestions on how to integrate their experiences in an online-support setting.

<u>**Part 1: Nurses' experiences and practices with supporting the process of behaviour change for cardiovascular prevention**</u>

For peer review only - http://bmjopen.bmj.com/site/about/guidelines.xhtml

Preconditions for behaviour change guidance

We identified three main themes, that both the Finnish and Dutch nurses regarded as preconditions for behaviour change guidance in their patients: establishing a relationship of trust, awareness and expectation management and appropriate timing and monitoring. Both groups of nurses explained what skills they used to realise these preconditions, showing subtle differences between the groups.

Establishing a relationship of trust

According to both the Finnish and Dutch nurses, the basis of behaviour change support lay in establishing a relationship of trust with the patient: developing over time a good nurse-patient relationship in which the individual felt at ease and respected and comfortable enough to open up about lifestyle and behaviour issues:

> *"For lifestyle change, for prevention, a relationship based on mutual trust is pivotal. It is good to have a many years' standing contact with people. Then you know what is going on in someone's life and in that, some kind of trust will grow that people really start believing what you are saying to them. And then, over time, people will start practising healthy behaviours that maybe they had no intention to follow, in the beginning"* (Dutch nurse 1)

Skills the nurses used to stimulate trust to grow, were personalising and tailoring their support to each patient, which included:

> *"And you need to get a good picture of the situation, so that you don't give the same information to everyone. That's of no use. You need to think what the central issues are for this patient. What are the things he or she seems to have resources for? What are the goals that the client sets? What is the client able to do, and with what kind of intensity? What will the time span be like? And I also ask my client directly that what kind of support does he or she wishes? I'm trying to offer what the client thinks he or she needs"* (Finnish nurse 1)

Interestingly, when further exploring these skills, the nurses expressed different preferences regarding the ideal mode of communication. The Dutch nurses emphasized the importance of face-to-face contact and in-person continuity to establish a good relationship. For Finnish nurses an initial face-to-face contact only seemed sufficient to establish a working relation:

> *"And here [refers to the HATICE platform] the initial contact and information session at the beginning is very important because I guess a sort of a relationship needs to be established here as well. In the same way. There are still people behind this platform."* (Finnish nurse 1)

For peer review only - http://bmjopen.bmj.com/site/about/guidelines.xhtml

Thereafter, they were comfortable with further phone or email contact and did not regard this as less personal than face-to-face contact. Email contact also had advantages:

> *"But sometimes this kind of communication online could be less complicated…than face to face."*(Finnish nurse 5)
>
> *"I have noticed in my work that some people prefer contacting me by e-mail and not by phone. [others agree] On the phone they might think that they are disturbing or it's a bad timing, but one can write an e-mail or something anytime."* (Finnish nurse 3)

Awareness and expectation management

A second precondition was awareness and expectation management: checking the patients' level of knowledge and expectations regarding prevention and personal cardiovascular risk. Nurses thought that most patients had considerable knowledge of cardiovascular disease prevention, especially in Finland, due to a long standing tradition in community based cardiovascular prevention (the North-Karelia project [27]). Nonetheless, both groups of nurses had the experience that people were not especially aware of their personal cardiovascular risk status:

> *"That's it, isn't it, for many people their health is not a concern yet. You can list them the facts, and they hear and read it everywhere, that it is unhealthy to have overweight and that they need to exercise more, but right now, they are not yet bothered by it."* (Dutch nurse 6)

Because of this lack of a sense of urgency, the nurses regarded the ability to educate their patients about consequences of health behaviours an essential skill of their profession. Once awareness and motivation had grown, people often had unrealistic expectations and the nurses needed to act as *"myth busters"* (Finnish nurse 4):

> *"And when we are, however, not able to offer the magic pills or wonder tricks, the clients may sometimes be disappointed when all I can suggest is these boring methods: diet and physical activity. And we cannot offer them a magic solution."* (Finnish nurse 4)

Often, once people were motivated to change their health behaviours, they also tended to set unrealistic goals, which the nurses then needed to bring back to realistic proportions:

> *"Start small. Do not make it too big. If you are obese, many people do not like it to go to the gym, they think the gym is only for lovely slim figures. You cannot convince them that that's not true. Therefore it is important: try things first yourself. What can you do with small steps at home by yourself, before going outside. You have to start liking exercise."* (Dutch nurse 3)

For peer review only - http://bmjopen.bmj.com/site/about/guidelines.xhtml

Lastly, the nurses actively prepared their patients for failures during the process of behaviour change, as these were seen as inevitable:

> *"I usually tell the patients that they're allowed to fail; but even so, they are invited to, and they should come to the appointments. So then we can check the situation again, and set a new goal if needed."* (Finnish nurse 1)

With the Finnish nurses, coaching on cardiovascular risk was also related to the potential for dementia prevention. They suggested that many patients feared dementia and lacked knowledge about the disease and treatment and prevention options, creating a stigma towards this condition. The nurses were aware of the link between cardiovascular disease and dementia, but felt they lacked sufficient knowledge and training to provide proper support:

> *"Well, we have not had the knowledge of reasons for dementia for that long. And these connections haven't been…the research is recent: well, at least more recent than the research about heart diseases." (Finnish nurse 5)*

They found that educating patients on the link between cardiovascular disease and dementia, would be a good starting point to raise awareness. Potentially, this could enhance motivation for CV prevention:

> *"What is good for the heart - and we know what's good for the heart – is also good for the brain but not everyone knows this. I think this link would be good to be aware of: you protect your heart but also the most important part of your body which is the brain."* (Finnish nurse 4)

Appropriate timing and monitoring

The third precondition mentioned by the nurses was appropriate timing and monitoring: providing professional support at appropriate times and monitoring the progress of the patient towards behaviour change. Regular follow-up appointments stimulated adherence and motivation:

> *"After three months, your plan fades away, your goal, your motivation."* (Dutch nurse 3).
>
> *"..that there is a possibility for follow-up. Usually it motivates people when someone looks after you: how are you progressing, no matter if the target is, for example, smoking cessation or increasing of physical activity."* (Finnish nurse 5)

Monitoring ensured that the nurses could support their patients when experiencing obstacles or failures, although this could be difficult:

For peer review only - http://bmjopen.bmj.com/site/about/guidelines.xhtml

> *"Disappointments also play a role. For example: a guy with diabetes, he quit smoking but then his sugar levels went up and he needed to start with insulin. How do you explain that [to him]? Well, I challenge you to keep his attitude up and to maintain his motivation."* (Dutch nurse 5)

When discussing monitoring lifestyle behaviours both nurse groups attributed themselves a supportive role putting the patient in charge, because lifestyle was seen as the personal domain of the patient. However, regarding the medical components of preventive care (control of hypertension, diabetes and hypercholesterolemia), the Dutch nurses attributed a more directive role to themselves and the medical practice to avoid mistakes and complications. In contrast, the Finnish nurses regarded their patients as capable of staying in charge and described themselves as mentors:

> *"It is also one of the nurse's responsibilities to be a contact person, support and a sort of mentor and also to refer the patient to a doctor if the nurse notices that something is going wrong."* (Finnish nurse 4)

Part 2: Integrating the nurses' strategies into an online-support setting

After having identified the preconditions for optimal behaviour change support and the skills nurses use in their current practices, we demonstrated the latest version of the HATICE internet-platform[26] and discussed how optimal online-support for CV prevention should be shaped and how they imagined providing online support. Both groups of nurses emphasised the importance of the aforementioned preconditions for optimal online support:

Establishing a relationship of trust

All nurses regarded the presence of a coach as essential to guarantee personal support. The Finnish nurses felt that online coaching could successfully establish a relationship of trust, provided that the coach was a real person:

> *"Because of this social interaction on this website [the HATICE platform], the participant has a familiar and friendly person [as a coach] and not just some distant virtual coach who is a stranger. [...] it's good that this combines the real-life person with the online contact, maybe it feels more comfortable and familiar [for the participant]."* (Finnish nurse 4)

An initial face-to-face consultation with the patient could strengthen the establishment of a good relationship. Overall, for the Finnish nurses, online support was an obvious step forward in innovating healthcare:

For peer review only - http://bmjopen.bmj.com/site/about/guidelines.xhtml

> *"Well at least I think that this is absolutely the trend [others nod and agree], that all the services will be at least partly available online for the patients. Partly like this [via internet] and partly with human contact. I think that it's an inevitable part of future."* (Finnish nurse 1)

In contrast, the Dutch nurses could not imagine the platform and coach fully substituting their personal guidance:

> *"The strength of our guidance is the personal contact we have with the patients. [...] that enables us to give them some subtle support and give them a small push into the right direction. To delegate all of that to an online coach just like that, that seems difficult to me. Then all personal contact will disappear."* (Dutch nurse 7)

Awareness and expectation management

All nurses regarded the internet-platform a suitable mean to raise awareness and increase health-literacy. Managing expectations related to online support was considered very important, because misunderstandings could arise more easily through this mean. Therefore, the coach should explain what could be expected from the platform and their support:

> *"Communication is very important in the beginning:, what is it we do and what do they expect from the goals."* (Dutch nurse 1)

Appropriate timing and monitoring

The nurses envisioned that online, the patient would be in charge of timing of support and monitoring of progress. The coach would have a reactive role, providing support in response to the patient's demand. However, the nurses felt the coach also needed to be proactive, in case people showed signs of losing motivation. This would require insight in people's platform activities:

> *"[...] the nurse can also see it [the diary] and check. If the participant fails to achieve the goals, the nurse can go back and check what might have been the problem."* (Finnish nurse 5)

Both groups thought the platform should be aligned to regular healthcare. The Finnish nurses envisioned that the online coach could work in the same fashion as the nurses currently did, targeting both lifestyle and medical components of their patient's health. The Dutch nurses stressed that not everybody would be able to self-manage, and therefore would be in need of different intensities of coaching:

For peer review only - http://bmjopen.bmj.com/site/about/guidelines.xhtml

> *"I think 2 or 3 types of platform users will arise: people who really get the concept of self-management (and start coaching themselves), people who need the coach (and give the coach access to their complete profile) and a group in between, alerting the coach if a goal has not been met."* (Dutch nurse 2)

While discussing this topic, they expressed ambivalence whether it was 'safe' to entrust their patients with self-management when it related to medical issues:

> *"I tend to think: if it is self-management, you shouldn't want to get yourself involved in that [medication use], you should leave that with the GP. On the other hand, if someone's blood pressure is constantly rising, then you do want to know which medication someone is taking, to get the complete picture. Because then you check whether there might be a problem in medication-use."* (Dutch nurse 3)

At the end of this discussion, for safety reasons, they concluded they preferred a platform focusing on lifestyle only, staying in control of medical issues within the GP practice.

For peer review only - http://bmjopen.bmj.com/site/about/guidelines.xhtml

DISCUSSION

Principal findings and interpretation

In this international focus group study, we identified three main themes that both the Finnish and Dutch nurses emphasised as most important preconditions for behaviour change support in cardiovascular prevention, and potentially, prevention of cognitive decline: (1) establishing a relationship of trust, (2) managing awareness and expectations and (3) appropriate timing and monitoring of the process of behaviour change. These preconditions were regarded as important also to provide optimal online support. The nurses stressed that a coach providing human support and integration with regular care were essential elements to achieve this. They expressed, however, different ideas on its implementation (**Figure 1**).

Figure 1. Schematic visualization of the main themes and their connections

As mentioned in the introduction, realising and maintaining health behaviour change is notoriously complex. This was confirmed by the nurses we interviewed, but their clinical experience provided us with clear preconditions for optimal behaviour change support. The nurses used slightly different approaches to fulfil these preconditions, both in their current practice and in their ideas on online support. To establish a relationship of trust, the Dutch nurses relied more on face-to-face contact than the Finnish nurses, which appeared to make them more sceptical about the effectiveness of online coaching. The Finnish nurses took a mainly supportive role in monitoring whereas Dutch nurses emphasised a more directive role for themselves and the general practice, with regard to medical aspects of preventive guidance. This different attitude towards patient' autonomy is of interest and may be influenced by different factors, including health care culture, the geographical factor, the nurse factor and the patient factor. Regarding health care culture, **Box 3** describes that, although aims of preventive care are very similar between Finland and the Netherlands, patient empowerment and patient autonomy have received more emphasis in Finland than in the Netherlands. The nurses' ideas about their own responsibilities and patient autonomy may be aligned to the way patient-autonomy is being shaped in the two healthcare systems as well as to the description of nurses' responsibilities in local cardiovascular risk management guidelines. The different attitudes on face-to-face contact can be further understood from the perspective of geography. Finland is a large but very sparsely populated country and the Netherlands are a very small but densely populated country. This has influenced current organisation and accessibility of care and likely also attitude towards care (see **Box 3**). In this light, the step towards online care is likely to be smaller for the Finnish nurses. Last, the differences between the patient populations' age may have also influenced our findings, since the nurses might see more potential for eHealth applications in younger patients and regard them as more autonomous in their health behaviours.

For peer review only - http://bmjopen.bmj.com/site/about/guidelines.xhtml

Our results concerning dementia prevention are very preliminary but of special interest. The Finnish nurses liked the idea of including cognitive health as a goal for cardiovascular preventive care, as dementia was regarded a growing public health problem and a combined approach could increase people's motivation to engage in behaviour change. However, the nurses felt they could not provide proper support, given their limited knowledge and training on one hand, and limited extent of final scientific evidence on the other.

Strengths and limitations

The HATICE project is novel in its aim to develop a generic innovative cardiovascular prevention strategy for older people that can be used across European healthcare systems, especially since it involves eHealth. Our qualitative research design enabled us to use the best practices of nurses experts in 'traditional' face-to-face cardiovascular preventive care to make recommendations for optimal health behaviour change support through novel internet-platforms. In qualitative research, international joint analyses are not common because of language barriers. To overcome these, we put much effort in the alignment of our research methodology. The frequent interactions and extensive meetings of the research teams enabled us to explore our findings in the context of the local health care systems. Following grounded theory methodology [25], we deliberately selected nurses that we regarded experts in cardiovascular preventive care. Our research also has some limitations that may have influenced our findings. Information on non-participation was limited. The Finnish nurses had on average more years of clinical experience with CV prevention than the Dutch nurses. The patient populations of the Finnish and Dutch nurses were not identical with respect to age. This both may have influenced our findings. However, since the content of clinical experience of both groups was very similar and both countries have similar aims for cardiovascular prevention, we deem the selection of these nurses appropriate for our research purpose. Since we only performed two focus groups, we cannot exclude that a wider range of views could have been collected. For example, one might expect that themes related to training and education requirements would have emerged more prominently from the discussions, but this issue only was mentioned with regard to cognitive health. A further limitation is that cognitive health was only discussed with the Finnish nurses. This issue should be elaborated further in future studies. The striking similarities in the principal themes found in both countries and the consistency of our findings with previous literature mitigates fears that our samples were too limited. Last, when reviewing a summary of our findings, the nurses confirmed that their experiences and views were reflected and did not add new ones, emphasising that the most relevant themes were captured.

Comparison with existing literature

The importance of a relationship of trust, clarifying patients expectations and providing personally tailored support where also main themes in other European qualitative studies on cardiovascular

For peer review only - http://bmjopen.bmj.com/site/about/guidelines.xhtml

preventive care with nurses or patients [28-32]. The positive attitude of the Finnish nurses on self-management of medical issues was consistent with another Finnish study about nurses' and physicians' perceptions on patient's responsibilities in self-care [33]. The reserved attitude of the Dutch nurses was also reflected in a survey among Dutch healthcare professionals, where 50% feared that patient's direct access to their medical record would cause misunderstandings and unnecessary anxiety[34]. A recent qualitative systematic review on nurses' experiences of facilitators and barriers of using telehealth also reported on both positive and negative attitudes of nurses towards telehealth. With regard to the nurse-patient relationship, nurses mentioned on the positive side that telehealth could improve trusting relationships and lower access to care. On the negative side nurses reported telehealth could lead to a loss of human contact. Differences in attitudes were not linked to local health care cultures[35]. Finally, the conviction of all nurses in our study that a coach was essential to complement the internet-platform, is supported by a meta-analysis we performed showing that internet-interventions combined with human support were more effective than 'stand-alone' interventions [36].

Implications for practice

Finnish and Dutch nurses have similar experiences with and views on supporting behaviour change for cardiovascular prevention, but use different practical approaches towards their patients. Including the maintenance of cognitive health as a goal of cardiovascular prevention can provide novel opportunities to frame health behaviour change for both prevention of dementia and CVD and might augment people's motivation for prevention, but this suggestion should be studied further. The nurses' experiences provide valuable directions for shaping online support in internet-platforms for cardiovascular self-management. This study also indicates that, when introducing new forms of preventive healthcare that involve patient self-management, like internet-platforms, local healthcare practices are to be taken into account to achieve optimal engagement.

For peer review only - http://bmjopen.bmj.com/site/about/guidelines.xhtml

ADDITIONAL INFORMATION

Contributors

Study design: CB, ER, EMvC, HS, FM and MK. Data acquisition: CB, MB and AR. Data analysis: CB, UA, MB and AR. Interpretation of results: CB, UA, MB, AR, FM, EMvC and JP. Drafting of the manuscript: CB. Critical revision of the manuscript for important intellectual content: all authors.

Funding

The research leading to these results has received funding from the European Union Seventh Framework Programme for the HATICE-project (FP7/2007-2013, grant agreement n° 305374) and the Multimodal preventive trials for Alzheimer´s Disease: towards multinational strategies – programme (MIND-AD: MIND-AD Academy of Finland (grant agreement n°291803), MIND-AD VTR, Kuopio University Hospital ((grant agreement n°5772815) and MIND-AD Academy of Sweden (Swedish Research Council, 529-2014-7503)) .

Competing interests

The authors have declared no competing interests.

Ethical approval

In the Netherlands, the study was presented to the medical ethics committee of the Academic Medical Centre in the Netherlands and a waiver was provided. In Finland, application for ethical approval nor a waiver were required. Written informed consent was obtained from all participants in both countries.

Data sharing statement

Data will not be made publicly available. Please contact the corresponding author for more information.

Acknowledgements

We thank all Finnish and Dutch nurses for their participation in the study, Suzanne Ligthart, Carin Miedema, Paulien Vermunt, Floor Rooskens (respectively discussion moderator, assistant-moderator and assistants in transcription and coding for the Dutch meetings), Lotta Salo and Ejja Pietilä (organisation and summary notes of the Finnish meeting) for their assistance to the study.

For peer review only - http://bmjopen.bmj.com/site/about/guidelines.xhtml

REFERENCES

1. Oliver D. 21st century health services for an ageing population: 10 challenges for general practice. *The British journal of general practice : the journal of the Royal College of General Practitioners* 2012;62(601):396-7. doi: 10.3399/bjgp12X653435 [published Online First: 2012/08/08]
2. Wu YT, Fratiglioni L, Matthews FE, et al. Dementia in western Europe: epidemiological evidence and implications for policy making. *The Lancet Neurology* 2016;15(1):116-24. doi: 10.1016/s1474-4422(15)00092-7 [published Online First: 2015/08/25]
3. de Bruijn RF, Bos MJ, Portegies ML, et al. The potential for prevention of dementia across two decades: the prospective, population-based Rotterdam Study. *BMC medicine* 2015;13:132. doi: 10.1186/s12916-015-0377-5 [published Online First: 2015/07/22]
4. Kivipelto M, Ngandu T, Laatikainen T, et al. Risk score for the prediction of dementia risk in 20 years among middle aged people: a longitudinal, population-based study. *The Lancet Neurology* 2006;5(9):735-41. doi: 10.1016/s1474-4422(06)70537-3 [published Online First: 2006/08/18]
5. Norton S, Matthews FE, Barnes DE, et al. Potential for primary prevention of Alzheimer's disease: an analysis of population-based data. *The Lancet Neurology* 2014;13(8):788-94. doi: 10.1016/s1474-4422(14)70136-x [published Online First: 2014/07/18]
6. Peters R, Beckett N, Forette F, et al. Incident dementia and blood pressure lowering in the Hypertension in the Very Elderly Trial cognitive function assessment (HYVET-COG): a double-blind, placebo controlled trial. *The Lancet Neurology* 2008;7(8):683-9. doi: 10.1016/s1474-4422(08)70143-1 [published Online First: 2008/07/11]
7. Leventhal H, Weinman J, Leventhal EA, et al. Health psychology: the search for pathways between behavior and health. *Annu Rev Psychol* 2008;59:477-505.
8. Rosenstock IM. The Health Belief Model and Preventive Health Behavior. *Health Education Monographs* 1974;2(4):354-86. doi: 10.1177/109019817400200405
9. Ajzen I. From Intentions to Actions: A Theory of Planned Behavior. In: Kuhl J, Beckmann J, eds. Action Control: From Cognition to Behavior. Berlin, Heidelberg: Springer Berlin Heidelberg 1985:11-39.
10. Bandura A. Self-efficacy: Toward a unifying theory of behavioral change. *Psychological Review* 1977;84(2):191-215. doi: 10.1037/0033-295X.84.2.191
11. Prochaska JO, DiClemente CC, Norcross JC. In search of how people change. Applications to addictive behaviors. *The American psychologist* 1992;47(9):1102-14. [published Online First: 1992/09/01]
12. Weinstein ND. The precaution adoption process. *Health Psychology* 1988;7(4):355-86. doi: 10.1037/0278-6133.7.4.355
13. Burke LE, Dunbar-Jacob JM, Hill MN. Compliance with cardiovascular disease prevention strategies: a review of the research. *Ann Behav Med* 1997;19(3):239-63.
14. Abraham C, Kelly MP, West R, et al. The UK national institute for health and clinical excellence public health guidance on behaviour change: A brief introduction. *Psychology, Health & Medicine* 2009;14(1):1-8. doi: 10.1080/13548500802537903
15. Busse R, Blümel M, Scheller-Kreinsen D, et al. Tackling chronic disease in Europe Strategies, interventions and challenges. *World Health Organization 2010, on behalf of the European Observatory on Health Systems and Policies* 2010;20
16. Bodenheimer TL, K.; Holman, H.; Grumbach, K. Patient Self-Management of Chronic Disease in Primary Care. *Jama* 2002;288(19):2469 - 75.
17. Lorig K, Holman H. Self-Management Education: History, Definition, Outcomes, and Mechanisms. *Ann Behav Med* 2003;26(1):1-7.
18. Griffiths F, Lindenmeyer A, Powell J, et al. Why are health care interventions delivered over the internet? A systematic review of the published literature. *Journal of medical Internet research* 2006;8(2):e10. doi: 10.2196/jmir.8.2.e10 [published Online First: 2006/07/27]
19. Bennett GG, Glasgow RE. The delivery of public health interventions via the Internet: actualizing their potential. *Annual review of public health* 2009;30:273-92. doi: 10.1146/annurev.publhealth.031308.100235 [published Online First: 2009/03/20]

For peer review only - http://bmjopen.bmj.com/site/about/guidelines.xhtml

20. Patnode CD, Evans CV, Senger CA, et al. U.S. Preventive Services Task Force Evidence Syntheses, formerly Systematic Evidence Reviews. Behavioral Counseling to Promote a Healthful Diet and Physical Activity for Cardiovascular Disease Prevention in Adults Without Known Cardiovascular Disease Risk Factors: Updated Systematic Review for the US Preventive Services Task Force. Rockville (MD): Agency for Healthcare Research and Quality (US) 2017.
21. Richard E, Jongstra S, Soininen H, et al. Healthy Ageing Through Internet Counselling in the Elderly: the HATICE randomised controlled trial for the prevention of cardiovascular disease and cognitive impairment. *BMJ Open* 2016;6(6):e010806. doi: 10.1136/bmjopen-2015-010806 [published Online First: 2016/06/12]
22. Glaser BG, Strauss AL. The discovery of grounded theory. *Aldine, Chicago* 1967
23. Strauss A, Corbin J. Grounded theory methodology. *Handbook of qualitative research* 1994:273-85.
24. Tong A, Sainsbury P, Craig J. Consolidated criteria for reporting qualitative research (COREQ): a 32-item checklist for interviews and focus groups. *International Journal for Quality in Health Care* 2007;19(6):349-57. doi: 10.1093/intqhc/mzm042
25. Foley G, Timonen V. Using Grounded Theory Method to Capture and Analyze Health Care Experiences. *Health Services Research* 2015;50(4):1195-210. doi: 10.1111/1475-6773.12275
26. Jongstra S, Beishuizen C, Andrieu S, et al. Development and Validation of an Interactive Internet Platform for Older People: The Healthy Ageing Through Internet Counselling in the Elderly Study. *Telemedicine journal and e-health : the official journal of the American Telemedicine Association* 2016 doi: 10.1089/tmj.2016.0066 [published Online First: 2016/07/28]
27. Puska P, Nissinen A, Tuomilehto J, et al. The community-based strategy to prevent coronary heart disease: conclusions from the ten years of the North Karelia project. *Annual review of public health* 1985;6:147-93. doi: 10.1146/annurev.pu.06.050185.001051 [published Online First: 1985/01/01]
28. Loon MS, van Dijk-de Vries A, van der Weijden T, et al. Ethical issues in cardiovascular risk management: Patients need nurses' support. *Nursing ethics* 2014;21(5):540-53. doi: 10.1177/0969733013505313 [published Online First: 2013/11/22]
29. Ligthart SA, van den Eerenbeemt KD, Pols J, et al. Perspectives of older people engaging in nurse-led cardiovascular prevention programmes: a qualitative study in primary care in the Netherlands. *The British journal of general practice : the journal of the Royal College of General Practitioners* 2015;65(630):e41-8. doi: 10.3399/bjgp15X683149 [published Online First: 2014/12/31]
30. Brobeck E, Bergh H, Odencrants S, et al. Primary healthcare nurses' experiences with motivational interviewing in health promotion practice. *Journal of clinical nursing* 2011;20(23-24):3322-30. doi: 10.1111/j.1365-2702.2011.03874.x [published Online First: 2011/10/25]
31. Boase S, Mason D, Sutton S, et al. Tinkering and tailoring individual consultations: how practice nurses try to make cardiovascular risk communication meaningful. *Journal of clinical nursing* 2012;21(17-18):2590-8. doi: 10.1111/j.1365-2702.2012.04167.x [published Online First: 2012/07/14]
32. Hornsten A, Lindahl K, Persson K, et al. Strategies in health-promoting dialogues--primary healthcare nurses' perspectives--a qualitative study. *Scandinavian journal of caring sciences* 2014;28(2):235-44. doi: 10.1111/scs.12045 [published Online First: 2013/04/19]
33. Jallinoja P, Absetz P, Kuronen R, et al. The dilemma of patient responsibility for lifestyle change: Perceptions among primary care physicians and nurses. *Scand J Prim Health Care* 2007;25(4):244-49. doi: 10.1080/02813430701691778
34. Krijgsman J, Peeters J, Burghouts A, et al. Between the spark and the flame: 2015 eHealth monitor. *Nictiz and the NIVEL Institute [Dutch Institute for Research in the Healthcare Sector]* 2015;October
35. Koivunen M, Saranto K. Nursing professionals' experiences of the facilitators and barriers to the use of telehealth applications: a systematic review of qualitative studies. *Scandinavian journal of caring sciences* 2018;32(1):24-44. doi: 10.1111/scs.12445 [published Online First: 2017/08/05]

For peer review only - http://bmjopen.bmj.com/site/about/guidelines.xhtml

36. Beishuizen CR, Stephan BC, van Gool WA, et al. Web-Based Interventions Targeting Cardiovascular Risk Factors in Middle-Aged and Older People: A Systematic Review and Meta-Analysis. *Journal of medical Internet research* 2016;18(3):e55. doi: 10.2196/jmir.5218 [published Online First: 2016/03/13]
37. Hämäläinen P, Reponen J, Winblad I. eHealth of Finland - Checkpoint 2008. 2009
38. Säkkinen S, Heino A, Häkkinen P, et al. Statistical yearbook on social welfare and health care 2015 - National Institute for Health and Welfare. 2016
39. Vuorenkoski L, Mladovsky P, Mossialos E. Finland: Health system review. *Health Systems in Transition* 2008;10(4):1-168.
40. Ministry of Public Health, Welfare and Sport - Healthcare in the Netherlands. 2016
41. Schäfer W, Kroneman M, Boerma W, et al. The Netherlands: Health system review *Health Systems in Transition* 2010;12(1):1-229.
42. Kringos D, Boerma W, Bourgueil Y, et al. The strength of primary care in Europe: an international comparative study. *The British journal of general practice : the journal of the Royal College of General Practitioners* 2013;63(616):e742-e50. doi: 10.3399/bjgp13X674422
43. http://www.kaypahoito.fi/web/english/guidelineabstracts/guideline?id=ccs00014 [Finnish Current Care Guideline on hypertension (2014)]. accessed 7-12-2016 2016.
44. http://www.kaypahoito.fi/web/english/guidelineabstracts/guideline?id=ccs00032 [Finnish Current Care Guideline on diabetes (2016)]. accessed 7-12-2016 2016.
45. http://www.kaypahoito.fi/web/english/guidelineabstracts/guideline?id=ccs00055 [Finnish Current Care Guideline on dyslilidaemias (2013)]. accessed 7-12-2016 2016.
46. Boomsma L, Lakerveld-Heyl K, Gorter K, et al. Landelijke Eerstelijns Samenwerkings Afspraak Diabetes mellitus type 2. *Huisarts Wet* 2006;49(8):418-24.
47. den Boer J. Protocollair CVRM: Nederlands Huisartsen Genootschap 2016.
48. Faber E, De Bont M, Beusmans G, et al. Landelijke Eerstelijns Samenwerkings Afspraak CVA. *Huisarts Wet* 2007;50(3):S5-S8.
49. http://www.kanta.fi/en [Website of the Finnish Patient Data Repository, where Finnish people can access their own medical record]. accessed 7-12-2016 2016.

For peer review only

For peer review only - http://bmjopen.bmj.com/site/about/guidelines.xhtml

1 **BOXES**

2

3 **Box 1:** the Finnish and Dutch primary care systems

	Finland	The Netherlands
Organisation	Primary care is delivered through public healthcare centres and through occupational health facilities[37-39]. Companies offer occupational health facilities to their employees, including both preventive and curative health services, delivered through semi-private healthcare centres that work with nurses in a similar fashion as the public centres.	General practices or small health care centres. In most general practices continuity of care is ensured by allocating the patient to one GP.
Main focus	Primary care has a strong position and important gatekeeper function. Health promotion and disease prevention are main focus of health care policy for decades.	Access to care for everyone and solidarity through medical insurance[40 41]. GPs are gatekeepers of the healthcare system and provide acute, chronic and preventive care.
Accessibility	Often, healthcare centres cover large geographical areas that are sparsely populated and often have shortage of staff, contributing to long waiting lists and lack of personal continuity of care. Due to these waiting lists, many employees go to their occupational health service instead.	Since the Netherlands are densely populated, people often live at short distance from their general practice. Access to the GP is efficient, there are no waiting lists[42].
Role of primary care nurses	Important position: nurses work in close collaboration with the GPs and have their own consulting hours to assess patients. Regarding cardiovascular prevention, they monitor patients with diabetes, hypertension and dyslipidaemia, as described in national guidelines[43-45].	Important position: since several decades, GPs have delegated tasks to practice nurses in chronic disease management. Currently, nurses provide a substantial part of cardiovascular risk management care, including diabetes care, following regional and national guidelines and work descriptions[9 46-48].
Patient autonomy and eHealth culture to date	The first European country to introduce a law (in 1993) defining the patient's right to access to all medical information and the right to autonomy (patient's informed consent for any medical treatment). All healthcare centres use electronic medical	Informed consent is ensured by law, but in daily practice, consent is often assumed and only explicitly discussed in case treatment options can have far-reaching consequences[41]. Almost all GPs use electronic medical records. Patients have the right to inspect their

For peer review only - http://bmjopen.bmj.com/site/about/guidelines.xhtml

	records. A national patient data repository is under development to provide patients complete access to their own electronic medical record[49]	medical records, but do not have complete access to them.

Box 2. Main topics discussed

Part 1

- Prevention of cardiovascular disease and dementia: attitude and experiences
- Good guidance of behaviour change
- Relationship with the patient

Part 2

- Attitude towards the internet-platform and online-support
- Role and responsibilities of the internet-coach
- Interaction with the patients online

Box 3. Key features of the HATICE internet-platform with coaching

- Patient-centred: the patient can login onto a personal portal to review and manage his/her personal cardiovascular risk profile
- Improving health knowledge: the patient can access educational modules about cardiovascular risk factors and lifestyle
- Goal setting and self-monitoring: the patient can set his/her own goals for behaviour change and monitor how he/she is doing by entering self-measurements or keeping a diary
- Coaching: the coach monitors the patient's self-management and they can communicate online through messages

For peer review only - http://bmjopen.bmj.com/site/about/guidelines.xhtml

1

For peer review only - http://bmjopen.bmj.com/site/about/guidelines.xhtml

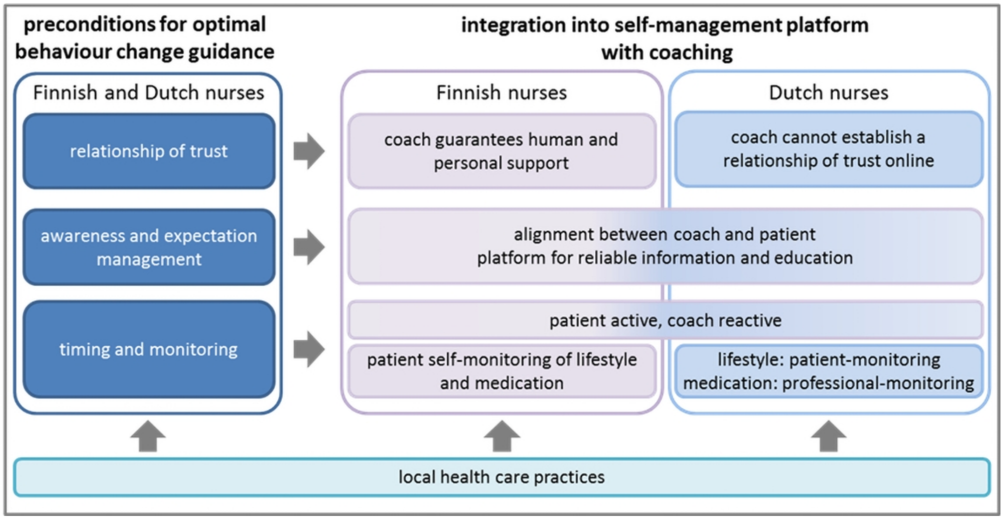

Caption: Figure 1. Schematic visualization of the main themes and their connections
Legend: Left, the three main preconditions for good behaviour change guidance in cardiovascular preventive care that both Finnish and Dutch nurses identified, are depicted. Right of this, it is shown how the Finnish and Dutch nurses suggest to realise these preconditions in the online setting. Since there were differences between the nurses this is depicted separately for the Finnish and Dutch nurses. Below it is shown that local health practices influenced both the preconditions (and their operationalization (not shown in figure but explained in results section)) and the integration into online support.

194x99mm (300 x 300 DPI)

APPENDIX 1 to 'Integrating nurses' experiences with supporting behaviour change for cardiovascular prevention into a self-management internet-platform in Finland and the Netherlands: a qualitative study'

Appendix 1: COREQ checklist

No.	Item	Explanation	Reported on page no.
Domain 1:Research team and reflexivity			
1	Interviewers	NL: Pols AJ and Ligthart S<br>FI: Rosenberg A	Not reported in manuscript
2	Credentials of the researchers involved in data collection and analysis	NL: Beishuizen CRL: MD PhD-student; Rooskens F: BSc; Lighthart S: MD, PhD-student; Pols AJ: prof; Moll van Charante EP: MD PhD<br>FI: Akenine U: PhD-student; Barbera M: PhD; Rosenberg A: PhD-student	Not reported in manuscript
3	Occupation of the researchers involved in data collection and analysis	NL: Beishuizen CRL: PhD-student; Rooskens F: medical student; Ligthart S: general practitioner in training, PhD-student; Pols AJ: professor in medical ethics; Moll van Charante EP: postdoc researcher, general practitioner<br>FI: Akenine U: PhD-student, research nurse; Barbera M: postdoc researcher; Rosenberg A: PhD-student	Not reported in manuscript
4	Gender	All researchers are female, except for Moll van Charante EP, who is male	Not reported in manuscript
5	Experience and training	See main text	p5
6	Relationship established	NL: there was an indirect relationship between research team and the partcipants, because the nurses had been involved in a previous research project of the research team in which 2 researchers (Ligthart S and Moll van Charante EP) were also involved<br>FI: no previous relationship established	Not reported in manuscript
7	Partcipant knowledge of the	NL: participants knew the professional background of the moderators	Not reported in

	interviewer	FI: participants knew the professional background of the moderator	manuscript
8	Interviewer characteristics	NL: Pols AJ conducts qualitative research on the ethics of use of technology and eHealth in medical care. Ligthart S conducts quantitative and qualitative research on cardiovascular prevention in older people<br><br>FI: Rosenberg A conducts research on prevention of dementia	Not reported in manuscript
Domain 2: Study design			
9	Methodological information and theory	See main text	P5 and p6
10	Sampling	See main text	P5
11	Method of approach	See main text	P5
12	Sample size	See main text	P5
13	Non-participation	See main text	P5
14	Setting of data collection	See main text	P5
15	Presence of non-participants	NL: Eric Moll- van Charante and Pim Happel were present as non-participating audience<br><br>FI: no others were present	Not reported in manuscript
16	Description of sample	See main text	P5 and p6
17	Interview guide	See main text	P6
18	Repeat interviews	Not performed	
19	Audio/visual recording	See main text	P6
20	Field notes	See main text	P6
21	Duration	See main text	P6

For peer review only - http://bmjopen.bmj.com/site/about/guidelines.xhtml

22	Data saturation	See main text	P7 and P15
23	Transcripts returned	Not performed	
Domain 3: analysis and findings			
24	Number of data coders	NL: 2<br>FI: 2	P6
25	Description of the coding tree	See main text (figure 1)	p14
26	Derivation of themes	See main text	P6
27	Software	No special qualitative software was used	
28	Participant checking	See main text	P7 and p15
29	Quotations presented	See main text	P8– p13
30	Data and findings consistent	See main text	P8 – p13
31	Clarity of major themes	See main text and figure 1	P8-p14
32	Clarity of minor themes	Within the groups, the Dutch and Finnish nurses shared opinions and experiences on most topics. Between the Dutch and Finnish groups, some interesting differences in opinions and experiences were identified. We choose therefore to focus on these differences when presenting our results, but not on diverse cases within the groups.<br><br>Minor themes were not discussed due to word limits	Not reported in manuscript

For peer review only - http://bmjopen.bmj.com/site/about/guidelines.xhtml

APPENDIX 2 to 'Integrating nurses' experiences with supporting behaviour change for cardiovascular prevention into a self-management internet-platform in Finland and the Netherlands: a qualitative study'

Appendix 2: Topic list

**FOCUS GROUP nurses part ONE cardiovascular prevention**		
**Overview part one**	**Topic (possible items)**	**Possible questions**
Aim of the meeting is to learn from the nurses' experiences in cardiovascular prevention: what are best practices to stimulate behaviour change, what is their attitude towards a prevention program for older people via the internet, how would they support people in lifestyle change through the internet?		
	**CVD prevention**	
	Activities on CVD prevention	Do you currently conduct activities on CVD prevention?
	Experiences / lessons learned	What are your experiences and lessons learned, especially regarding giving 'medical' guidance versus giving lifestyle guidance and regarding behavior change?
	**Dementia prevention**	In the last decade, we got more and more indications from neurological research that risk factors for cardiovascular disease are also risk factors for dementia. So, possibly, reducing cardiovascular risk may also postpone or prevent dementia.
	Attitude towards dementia prevention	What is your attitude / your ideas regarding dementia prevention?
	Awareness of dementia risk and compliance to lifestyle change	Most people are not yet aware of the association between CV risk and dementia, but do seem to be very afraid of dementia. Do you think that more awareness would enhance compliance/adherence to lifestyle change? What are your ideas on this?
	**Relationship with participants and regular healthcare system**	
	Guiding lifestyle change	Which factors could contribute to good guidance of lifestyle change?
	Relationship with patient	Which factors could contribute to a good relationship with your patient?
	Relation with GP and regular practice assistant	How should the platform coach link with the patients' GP and regular practice assistant/nurse (the regular healthcare system)?

For peer review only - http://bmjopen.bmj.com/site/about/guidelines.xhtml

	Attitude towards internet intervention	What is your attitude towards a prevention program via the internet (with support from a coach)?
		How should the platform coach link with the patients' GP and regular practice assistant/nurse (the regular healthcare system)?
**FOCUS GROUP nurses part TWO The platform**		
Assistant moderator explains about HATICE and platform by showing the powerpoint with screen shots of the platform		
	**Information required for support**	Imagine yourself being a coach using the internet-platform:
		Which kind of information regarding the participants do you need to be able to support them?
	**Role and responsibilities**	
	Responsibility goal setting	Who is responsible for goal setting? (capability of patients)
	Role in lifestyle groups	How do you see your role in creating lifestyle groups and how can participation be encouraged?
	**Interaction with participants**	
	Experience with motivational interviewing	Do you have experience with motivational interviewing, how could this technique be used by the coach?
	Frequency of contact	How often would you like to have contact with your patient?
	Mode of communication	Which mode of communication do you prefer? (phone, skype +/- webcam, email, chat…)
	Network of support	What should be the role of the participant network of support in lifestyle change and how can this network be engaged?
	Alerts / reminders	Do you like to receive automatic alerts/reminders when patients have alarming values or did not log-on?
Assistant moderator and moderator verify key messages from focus group		
Moderator thanks nurses		

For peer review only - http://bmjopen.bmj.com/site/about/guidelines.xhtml

BMJ Open

Integrating nurses' experiences with supporting behaviour change for cardiovascular prevention into a self-management internet-platform in Finland and the Netherlands: a qualitative study

Journal:	*BMJ Open*
Manuscript ID	bmjopen-2018-023480.R2
Article Type:	Research
Date Submitted by the Author:	13-Mar-2019
Complete List of Authors:	Beishuizen, Cathrien; Academic Medical Center, General Practice<br>Akenine, Ulrika; Karolinska Institutet and Karolinska University Hospital, Division of Clinical Geriatrics , Department of Neurobiology, Care Sciences and Society<br>Barbera, Mariagnese; University of Eastern Finland, Clinical Medicine/Neurology<br>Rosenberg, Anna; University of Eastern Finland, Clinical Medicine/Neurology<br>Fallah Pour, Mandana; Karolinska Institutet, Division of Occupational Therapy, Department of Neurobiology, Care Sciences and Society<br>Richard, Edo; Academic Medical Centre, University of Amsterdam, Neurology; Radboud University Medical Center, Department of Neurology, Donders Institute for Brain, Cognition and Behaviour<br>Soininen, Hilkka; University of Eastern Finland, Clinical Medicine/Neurology<br>Mangialasche, Francesca; Karolinska Institutet, Division of Geriatric Epidemiology<br>Kivipelto, Miia; Karolinska Institutet, Division of Geriatric Epidemiology; University of Eastern Finland, Clinical Medicine/Neurology<br>Pols, A.; AMC, Department of General Practice, Section of Medical Ethics,<br>Moll van Charante, Eric; Academic Medical Center, General Practice
**Primary Subject Heading**:	Cardiovascular medicine
Secondary Subject Heading:	General practice / Family practice, Health services research, Nursing, Patient-centred medicine, Qualitative research
Keywords:	QUALITATIVE RESEARCH, PRIMARY CARE, Nurse-led care, eHealth, Cardiovascular prevention, Dementia prevention

SCHOLARONE™
Manuscripts

Integrating nurses' experiences with supporting behaviour change for cardiovascular prevention into a self-management internet-platform in Finland and the Netherlands: a qualitative study

Authors

Beishuizen CRL[1*], Akenine U[2], Barbera M[3], Rosenberg A[3], Fallahpour M[4], Richard E[5,6], Soininen H[3,7], Mangialasche F[8], Kivipelto M[2, 3,9], Pols AJ[10] and Moll van Charante EP[1]

*corresponding author

address: Department of General Practice
Amsterdam UMC/University of Amsterdam
Meibergdreef 9, 1100 DD
Amsterdam, Netherlands

email: c.r.beishuizen@amc.uva.nl

phone: +31 6 16359453

Affiliations

[1] Department of General Practice, Amsterdam UMC/University of Amsterdam, Amsterdam, The Netherlands
[2] Division of Clinical Geriatrics, Department of Neurobiology, Care Sciences and Society, Karolinska Institutet, and Karolinska University Hospital, Stockholm, Sweden
[3] Institute of Clinical Medicine/Neurology, University of Eastern Finland, Kuopio, Finland
[4] Department of Neurobiology Care Sciences and Society, Division of Occupational Therapy, Karolinska Institutet, Huddinge, Sweden
[5] Department of Neurology, Amsterdam UMC/University of Amsterdam, Amsterdam, The Netherlands
[6] Department of Neurology, Donders Institute for Brain, Cognition and Behaviour, Radboud University Medical Center, Nijmegen, The Netherlands
[7] Neurocenter, Neurology, Kuopio University Hospital, Kuopio, Finland.
[8] Aging Research Center, Karolinska Institutet and Stockholm University, Stockholm, Sweden
[9] Chronic Disease Prevention Unit, National Institute for Health and Welfare, Helsinki,, Finland
[10] Section of Medical Ethics, Department of General Practice, Amsterdam UMC/University of Amsterdam, Amsterdam, The Netherlands

Word count

Abstract: 263

Total text (main text and quotations): 3,688

Main text only: 2,591

Quotations only: 1,097

For peer review only - http://bmjopen.bmj.com/site/about/guidelines.xhtml

Abstract

Objectives: Global ageing is linked to an increased burden of cardiovascular disease (CVD) and dementia, which calls for better prevention strategies. Self-management and eHealth applications are regarded as promising strategies to support prevention. The aim of this study was to explore nurses' best practices concerning behaviour change guidance for cardiovascular (CV) prevention in order to learn how to optimally integrate them into a coach-supported internet-platform for CV self-management.

Design: Qualitative focus group study in Finland and the Netherlands. Discussions were audiotaped and transcribed. Data were thematically analysed following principles of grounded theory.

Setting: Dutch and Finnish primary care settings.

Participants: Six Finnish and seven Dutch primary care nurses with experience in CV prevention

Results: Similar best practices were found in both countries and comprised of: (1) establishing a relationship of trust, (2) managing awareness and expectations, and (3) appropriate timing and monitoring of the process of behaviour change. However, the Finnish and Dutch nurses used different approaches for accomplishment of these practices, which was reflected in their recommendations for online-support. Both groups emphasised that online-support should be combined with human-support and integrated into regular care. Finnish nurses had more confidence in patient self-management and remote communication than Dutch nurses, who emphasised the importance of face-to-face contact and preferred to keep control of medical aspects of prevention.

Conclusions: Differences in Dutch and Finnish's nurses' practices for supporting CV prevention appear to reflect their local healthcare practices, which should be taken into account when designing internet-platforms for health self-management. Including cognitive health as a goal of CV prevention might stimulate motivation for health behaviour change.

Keywords: cardiovascular prevention, dementia prevention, behaviour change, eHealth, primary care, nurse-led care, qualitative research

Strengths and limitations of this study

- This international focus group study directly compares best practices of Finnish and Dutch primary care nurses in cardiovascular prevention
- Language barriers were overcome by closely aligning our research methodologies, multiple iterations in the analysis and extensive meetings between the research teams
- Our original approach, following grounded theory, enabled us to learn from the best practices of nurse experts in 'traditional' face-to-face cardiovascular preventive care and integrate these

For peer review only - http://bmjopen.bmj.com/site/about/guidelines.xhtml

into recommendations for optimal support for health behaviour change through novel eHealth applications

- Since our samples and number of focus groups were limited, we are aware that our findings are not exhaustive, especially since we found that local health care practices substantially impacted the study results
- Due to the exploratory nature of our study, our findings are preliminary and might be influenced by differences in nurses' clinical experience and in the ages of their patient populations

For peer review only

For peer review only - http://bmjopen.bmj.com/site/about/guidelines.xhtml

Manuscript

INTRODUCTION

Global ageing places an increasing demand on healthcare systems, partially due to the absolute rise in cardiovascular disease (CVD) and dementia cases [1 2]. As these disorders share a number of risk factors, effective cardiovascular (CV) prevention could also lead to the prevention of dementia [3-6]. CV prevention requires health behaviour change, the process of "initiating and maintaining behaviours that reduce health risks and control existent chronic disease"[7]. In CV prevention, core behaviours consist of a healthy lifestyle (healthy diet, sufficient physical activity and non-smoking) and adherence to medication. Although the processes supporting health behaviour change have been theorised extensively[8-12], putting them into practice remains a challenge[13 14] and novel, more effective, approaches are needed[15]. Two strategies of current interest are self-management and eHealth. In self-management, the individual, instead of the healthcare professional, takes the lead in the management of his/her risk factors, and therefore in behaviour change[16 17]. eHealth applications are attractive because of their wide reach and their potential to support self-management because of their suitability for health education, interactivity and monitoring[18 19]. Although researchers and policymakers have high expectations of eHealth and self-management, more intensive face-to-face interventions still achieve better results than eHealth applications[20]. To learn how self-management and behaviour change could be best stimulated and maintained online, we consulted 'experts in the field', i.e. nurses experienced in health behaviour change in the context of CV prevention.

This project is part of the Healthy Ageing Through Internet Counselling in the Elderly (HATICE) study, which includes a European randomised controlled trial testing a coach-supported internet-platform for self-management of cardiovascular risk factors in older people to prevent CVD and cognitive decline [21]. In an international focus group study, we aimed to (1) explore nurses' best practices concerning behaviour change guidance for cardiovascular prevention, including the potential for dementia prevention, and (2) learn how to integrate their practices into a coach-supported internet-platform (the online-support setting). This study took place in Finland and the Netherlands, two of the three countries that participated in the HATICE-study. Since the HATICE project aims to develop an internet-platform that is implementable across all European healthcare systems, we also explored the influence of local healthcare practices.

For peer review only - http://bmjopen.bmj.com/site/about/guidelines.xhtml

METHODS

Design

We performed an international qualitative focus group study following principles of grounded theory[22 23]. The COREQ-checklist is included for full methodological information (**Appendix 1**)[24].

Participants and setting

For sampling, we followed the grounded theory methodology of studying a health care practice by consulting field experts [25]. In this perspective, Finnish and Dutch primary care nurses experienced in CV preventive care were considered most eligible for this study and selective purposive samples were obtained. In Finland, occupational healthcare nurses were recruited because of their important role in preventive CV care (please see **Box 1** for a description of Finnish and Dutch primary health care systems and the position of Finnish occupational health). Fourteen nurses working in a semi-private healthcare centre in Kuopio (Eastern Finland) were invited by email and telephone and six female nurses (43%) consented to participate. Being occupational health nurses, they cared mostly for patients of working age. Duration of clinical experience with CV prevention ranged from 2 to 35 years. In the Netherlands, we recruited primary care nurses experienced in cardiovascular risk management. A group of 32 nurses experienced in CV preventive care working in general practices in two urban areas in the centre of the Netherlands was invited by email and telephone. Seven female nurses (22%) consented to participate. The unanimous reason for non-participation by Finnish and Dutch nurses was lack of time. The Dutch participating nurses cared for patients of all ages. Duration of clinical experience with CV prevention ranged from 3 to 11 years. **Table 1** contains further characteristics of the nurses.

Box 1. The Finnish and Dutch primary care systems

Table 1. Characteristics of the participating Finnish and Dutch nurses

Nr	Country*	Age	Training	Typ of CVD prevention	Internet use at work
**1**	FI	55	occupational health nurse	prim/sec prev	email, guideline use, referral, patient contact
**2**	FI	42	occupational health nurse	prim/sec prev	email, guideline use, referral
**3**	FI	25	occupational health nurse	prim/sec prev	email, guideline use, referral, patient contact
**4**	FI	45	occupational health nurse	prim/sec prev	email, guideline use, referral, patient contact
**5**	FI	49	occupational health nurse	prim/sec prev	email, guideline use, referral, patient contact
**6**	FI	60	occupational health nurse	prim/sec prev	guideline use, patient contact

For peer review only - http://bmjopen.bmj.com/site/about/guidelines.xhtml

**1**	NL	43	general nurse, practice nurse[a]	prim/sec prev	email, guideline use, referral, patient contact
**2**	NL	49	practice nurse	prim/sec prev	email, guideline use, referral, patient contact
**3**	NL	51	practice nurse	prim prev	email, guideline use, referral, patient contact
**4**	NL	53	general nurse, practice nurse	prim/sec prev	email, guideline use, referral
**5**	NL	42	practice nurse	sec prev	email, guideline use, referral, patient contact
**6**	NL	45	general nurse, practice nurse[b]	prim/sec prev	email, guideline use, referral, patient contact
**7**	NL	65	general nurse, practice nurse	prim/sec prev	email, guideline use, referral, patient contact

*abbreviations: CVD = cardiovascular disease, FI = Finland, prim = primary, sec = secondary, prev = prevention, NL = the Netherlands, DM = diabetes mellitus, COPD = chronic obstructive pulmonary disease

[a] practice nurse: received specific nursing training to work in general practice
[b] general nurse: received general nursing training to work as a hospital-based general nurse

The study was presented to the medical ethics committee of the Academic Medical Centre in the Netherlands and a waiver was provided. In Finland, neither application for ethical approval nor a waiver was required. All participants provided written informed consent.

Data collection

We conducted one focus group in the Netherlands (autumn 2013) and one in Finland (December 2015). We regarded focus groups as the most appropriate method to answer our research aims, because they enabled us to explore the experiences and attitudes of the nurses as thoroughly as possible, as the nurses could directly respond to each other's opinions and develop their ideas through the discussion. In each country, an experienced focus group moderator chaired the sessions, while an assistant-moderator noted non-verbal communication and summarised the discussions. The discussion was conducted using a topic list as a flexible guide (**Box 2** and **Appendix 2**). After the Dutch session, the topic list was refined for the Finnish focus group, to further explore the following topics: the nurse-patient relationship, attitudes towards eHealth applications, shaping optimal online support, the role of the nurse in this versus the role of the patient, and dementia prevention. The moderators asked open-ended questions, following principles of grounded theory, to inductively approach the data. Both moderators first asked the nurses about their activities in cardiovascular prevention and how they supported their patients in the process of behaviour change (**Part 1** of the focus group). The Finnish moderator also asked the nurses about their experiences with prevention of dementia. In **Part 2** of the focus group, the HATICE internet-platform was presented (**Box 3**, a full description of the platform is reported elsewhere [26]) and the nurses were asked how they would optimally support their patients in an

For peer review only - http://bmjopen.bmj.com/site/about/guidelines.xhtml

online setting. Both sessions lasted approximately two hours. The discussions were audio-recorded and transcribed.

Box 2. Main topics discussed

Box 3. Key features of the HATICE internet-platform with coaching

Coding and analysis

In each country, two researchers coded and thematically analysed the transcripts following principles of grounded theory [22 23]. Themes were derived inductively from the data and were not hypothesised beforehand. Open coding and identification of initial themes was first performed by the two researchers independently. Thereafter, codes and themes were compared. Dissimilarities were discussed until consensus was reached. Initial theme structure was then discussed with the senior researchers involved. In Finland, since the researchers were not Finnish native speakers, the transcript was translated into English and cross-checked by the Finnish focus group moderator, who was a Finnish native and fluent English speaker. In this way, the complete analysis of the Finnish data could be performed in English. After the initial analysis performed locally, themes and corresponding quotations of the Dutch sessions were also translated into English. The two research teams then had two meetings to discuss the structure of main themes and categories. The analysis-phase[22 23] was an iterative process, during which the researchers from both teams repeatedly returned to their data-files to add, merge and refine themes, until a definite theme structure was agreed on by all authors. During the iterative analysis-phase, the researchers discussed the themes and alternatives, and it was proposed that the local health care context could influence the differences found between caring styles of the two groups of nurses. Therefore, the research teams introduced their local health care systems (**Box 3**) to each other and these insights were used in further interpretation of the findings. A summary of the final conclusions was returned to the participants for feedback.

Patient and public involvement

Patients were not involved in the design of this substudy of HATICE. However, patients were involved in the development of the HATICE eHealth application by means of focus groups with the projected target population of the HATICE eHealth application and by means of consulting patient organisations (Dutch Heart Foundation and Dutch and Finnish Alzheimer Association)[26]. Results of this substudy were disseminated to the participants by means of a written summary.

RESULTS

We analysed the data from **Part 1** (the nurses' experiences and practices with supporting the process

For peer review only - http://bmjopen.bmj.com/site/about/guidelines.xhtml

of behaviour change for cardiovascular prevention, including the potential for dementia prevention) (Part 1) and **Part 2** (the nurses' suggestions on how to integrate their experiences in an online-support setting, stimulated by a demonstration of the HATICE-platform[26]) together, jointly informing the identification of 3 main themes. The themes can be understood as the nurses' preconditions for effective behaviour change guidance in their patients: establishing a relationship of trust; awareness and expectation management; and appropriate timing and monitoring. These were regarded as being equally important in 'off-line' and 'on-line' health care. Below, they are reported separately in relation to **Parts 1** and **2**, to distinguish the nurses' clinical experiences and practices in current health care settings from their recommendations for optimal online support.

Part 1: Nurses' experiences and practices with supporting the process of behaviour change for cardiovascular prevention

Preconditions for effective behaviour change guidance

Establishing a relationship of trust

According to both the Finnish and Dutch nurses, the basis of behaviour change support lay in establishing a relationship of trust with the patient, i.e. developing a good nurse-patient relationship over time, in which the individual felt at ease and respected and comfortable enough to open up about lifestyle and behaviour issues:

"For lifestyle change, for prevention, a relationship based on mutual trust is pivotal. It is good to have long-standing contact with people. Then you know what is going on in someone's life and in that, some kind of trust will grow, so that people really start believing what you are saying to them. And then, over time, people will start practising healthy behaviours that maybe they had no intention to follow, in the beginning" (Dutch nurse 1)

The nurses reported personalising and tailoring their support to each patient as skills used in order to stimulate trust. For example:

"And you need to get a good picture of the situation, so that you don't give the same information to everyone. That's of no use. You need to think what the central issues are for this patient. What are the things he or she seems to have resources for? What are the goals that the client sets? What is the client able to do, and with what kind of intensity? What will the time span be like? And I also ask my client directly what kind of support he or she would like. I try to offer what the client thinks he or she needs" (Finnish nurse 1)

Interestingly, when further exploring these skills, the nurses expressed different preferences regarding the ideal mode of communication. The Dutch nurses emphasized the importance of repeated face-to-

For peer review only - http://bmjopen.bmj.com/site/about/guidelines.xhtml

face contact and in-person continuity to establish a good relationship. For Finnish nurses an initial face-to-face contact only seemed sufficient to establish a working relationship:

"And here [refers to the HATICE platform] the initial contact and information session at the beginning is very important because I guess a sort of a relationship needs to be established here as well. In the same way. There are still people behind this platform." (Finnish nurse 1)

Thereafter, they were comfortable with further phone or email contact and did not regard this as less personal than face-to-face contact. Email contact was also considered to have advantages:

"But sometimes this kind of online communication could be less complicated…than face to face."(Finnish nurse 5)

"I have noticed in my work that some people prefer contacting me by e-mail and not by phone. [Others agree] On the phone they might think that they are disturbing me or that it's bad timing, but one can write an e-mail or something anytime." (Finnish nurse 3)

Awareness and expectation management

A second precondition was awareness and expectation management: checking the patients' level of knowledge and expectations regarding prevention and personal cardiovascular risk. Nurses thought that most patients had considerable knowledge of cardiovascular disease prevention, especially in Finland, due to a long standing tradition of community based cardiovascular prevention (the North-Karelia project [27]). Nonetheless, both groups of nurses had experienced that people were not especially aware of their personal cardiovascular risk status:

"That's it, isn't it. For many people, their health is not a concern yet. You can list them the facts, and they hear and read it everywhere, that it is unhealthy to be overweight and that they need to exercise more, but right now, they are not yet bothered by it." (Dutch nurse 6)

Because of this lack of a sense of urgency, the nurses regarded the ability to educate their patients about the consequences of health behaviours as an essential skill of their profession. Once awareness and motivation had grown, people often had unrealistic expectations and the nurses needed to act as *"myth busters"* (Finnish nurse 4):

"And when we are, however, not able to offer the magic pills or wonder tricks, the clients may sometimes be disappointed when all I can suggest is these boring methods: diet and physical activity. And we cannot offer them a magic solution." (Finnish nurse 4)

For peer review only - http://bmjopen.bmj.com/site/about/guidelines.xhtml

Often, once people were motivated to change their health behaviours, they also tended to set unrealistic goals, which the nurses then needed to bring back to realistic proportions:

> *"Start small. Do not make it too big. If you are obese, many people do not like it to go to the gym, they think the gym is only for lovely slim figures. You cannot convince them that that's not true. Therefore, it is important: try things first yourself. What can you do with small steps at home by yourself, before going outside? You have to start liking exercise."* (Dutch nurse 3)

Lastly, the nurses actively prepared their patients for failures during the process of behaviour change, as these were seen as inevitable:

> *"I usually tell the patients that they're allowed to fail; but even so, they are invited to, and they should come to the appointments. So then we can check the situation again, and set a new goal if needed."* (Finnish nurse 1)

With the Finnish nurses, coaching on cardiovascular risk was also related to the potential for dementia prevention. They suggested that many patients feared dementia and lacked knowledge about the disease and treatment and prevention options, creating a stigma towards this condition. The nurses were aware of the link between cardiovascular disease and dementia, but felt they lacked sufficient knowledge and training to provide proper support:

> *"Well, we have not had knowledge of the reasons for dementia for that long. And these connections haven't been…the research is recent: well, at least more recent than the research about heart diseases." (Finnish nurse 5)*

They found that educating patients on the link between cardiovascular disease and dementia, would be a good starting point to raise awareness. Potentially, this could enhance motivation for CV prevention:

> *"What is good for the heart - and we know what's good for the heart – is also good for the brain, but not everyone knows this. I think this link would be good to be aware of: you protect your heart but also the most important part of your body which is the brain."* (Finnish nurse 4)

Appropriate timing and monitoring

The third precondition mentioned by the nurses was appropriate timing and monitoring: providing professional support at appropriate times and monitoring the progress of the patient towards behaviour change. Regular follow-up appointments stimulated adherence and motivation:

> *"After three months, your plan fades away, your goal, your motivation."* (Dutch nurse 3).

> *"..that there is a possibility for follow-up. Usually it motivates people when someone looks after you: how are you progressing, no matter if the target is, for example, smoking cessation or increasing physical activity."* (Finnish nurse 5)

For peer review only - http://bmjopen.bmj.com/site/about/guidelines.xhtml

Monitoring ensured that the nurses could support their patients when they experienced obstacles or failures, although this could be difficult:

"Disappointments also play a role. For example: a guy with diabetes, he quit smoking but then his sugar levels went up and he needed to start with insulin. How do you explain that [to him]? Well, I challenge you to keep his attitude up and to maintain his motivation." (Dutch nurse 5)

When discussing monitoring lifestyle behaviours, both nurse groups attributed themselves a supportive role, putting the patient in charge, because lifestyle was seen as the patient's personal domain. However, regarding the medical components of preventive care (control of hypertension, diabetes and hypercholesterolemia), the Dutch nurses attributed a more directive role to themselves and the medical practice to avoid mistakes and complications. In contrast, the Finnish nurses regarded their patients as capable of staying in charge and described themselves as mentors:

"It is also one of the nurse's responsibilities to be a contact person, support and a sort of mentor, and also to refer the patient to a doctor if the nurse notices that something is going wrong." (Finnish nurse 4)

Part 2: Integrating the nurses' strategies into an online-support setting

Establishing a relationship of trust

All of the nurses regarded the presence of a coach as being essential for guaranteeing personal support. The Finnish nurses felt that online coaching could successfully establish a relationship of trust, provided that the coach was a real person:

"Because of this social interaction on this website [the HATICE platform], the participant has a familiar and friendly person [as a coach] and not just some distant virtual coach who is a stranger. […] it's good that this combines the real-life person with the online contact, maybe it feels more comfortable and familiar [for the participant]." (Finnish nurse 4)

An initial face-to-face consultation with the patient could strengthen the establishment of a good relationship. Overall, for the Finnish nurses, online support was an obvious step forward in innovating healthcare:

"Well at least I think that this is absolutely the trend [others nod and agree], that all the services will be at least partly available online for the patients. Partly like this [via internet] and partly with human contact. I think that it's an inevitable part of the future." (Finnish nurse 1)

In contrast, the Dutch nurses could not imagine the platform and coach fully substituting their personal guidance:

For peer review only - http://bmjopen.bmj.com/site/about/guidelines.xhtml

"The strength of our guidance is the personal contact we have with the patients. […] that enables us to give them some subtle support and give them a small push in the right direction. To delegate all of that to an online coach just like that, that seems difficult to me. Then all personal contact will disappear." (Dutch nurse 7)

Awareness and expectation management

All nurses regarded the internet-platform a suitable means to raise awareness and increase health-literacy. Managing expectations related to online support was considered very important, because misunderstandings could arise more easily through this method. Therefore, the coach should explain what could be expected from the platform and their support:

"Communication is very important in the beginning: what it is we do, and what do they expect from the goals." (Dutch nurse 1)

Appropriate timing and monitoring

The nurses envisioned that online, the patient would be in charge of timing of support and monitoring of progress. The coach would have a reactive role, providing support in response to the patient's demand. However, the nurses felt the coach also needed to be proactive, in case people showed signs of losing motivation. This would require insight into people's activities on the platform:

"[…] the nurse can also see it [the diary] and check. If the participant fails to achieve the goals, the nurse can go back and check what might have been the problem." (Finnish nurse 5)

Both groups thought the platform should be aligned to regular healthcare. The Finnish nurses envisioned that the online coach could work in the same fashion as the nurses currently did, targeting both lifestyle and medical components of their patient's health. The Dutch nurses stressed that not everybody would be able to self-manage, and therefore would be in need of different intensities of coaching:

"I think 2 or 3 types of platform users will arise: people who really get the concept of self-management (and start coaching themselves), people who need the coach (and give the coach access to their complete profile) and a group in-between, alerting the coach if a goal has not been met." (Dutch nurse 2)

While discussing this topic, they expressed ambivalence as to whether it was 'safe' to entrust their patients with self-management when it related to medical issues:

"I tend to think: if it is self-management, you shouldn't want to get yourself involved in that [medication use], you should leave that to the GP. On the other hand, if someone's blood pressure is constantly rising, then you do want to know which medication someone is taking, to get the complete picture. Because then you check whether there might be a problem in medication-use." (Dutch nurse 3)

For peer review only - http://bmjopen.bmj.com/site/about/guidelines.xhtml

At the end of this discussion, for safety reasons, they concluded that they preferred a platform focusing on lifestyle only, leaving medical issues within the control of the GP practice.

For peer review only

For peer review only - http://bmjopen.bmj.com/site/about/guidelines.xhtml

DISCUSSION

Principal findings and interpretation

In this international focus group study, we identified three main themes that both the Finnish and Dutch nurses emphasised as the most important preconditions for effective behaviour change support in cardiovascular prevention, and potentially, prevention of cognitive decline: (1) establishing a relationship of trust, (2) managing awareness and expectations and (3) appropriate timing and monitoring of the process of behaviour change. These preconditions were also regarded as important for providing optimal online support. The nurses stressed that a coach providing human support, and integration with regular care, were essential elements to achieve this. They expressed, however, different ideas on their implementation (**Figure 1**).

Figure 1. Schematic visualisation of the main themes and their connections

As mentioned in the introduction, making and maintaining health behaviour change is notoriously complex. This was confirmed by the nurses we interviewed, but their clinical experience provided us with clear preconditions for optimal behaviour change support. The nurses used slightly different approaches to achieve these preconditions, both in their current practice and in their ideas regarding online support. To establish a relationship of trust, the Dutch nurses relied more on face-to-face contact than the Finnish nurses, which appeared to make them more sceptical about the effectiveness of online coaching. The Finnish nurses took a mainly supportive role in monitoring, whereas Dutch nurses emphasised a more directive role for themselves and the general practice, with regard to medical aspects of preventive guidance. This different attitude towards patient autonomy is of interest and may be influenced by different factors, including healthcare culture, geographical factors, nurse factors, and patient factors. Regarding health care culture, as described in **Box 3**, although aims of preventive care are very similar between Finland and the Netherlands, patient empowerment and patient autonomy have received more emphasis in Finland than in the Netherlands. The nurses' ideas about their own responsibilities and patient autonomy may be aligned with the way patient-autonomy is being shaped in the two healthcare systems as well as with the description of nurses' responsibilities in local cardiovascular risk management guidelines. The different attitudes about face-to-face contact can be further understood from a geographical perspective. Finland is a large but very sparsely populated country and the Netherlands is a very small but densely populated country. This has influenced current organisation and accessibility of care, and probably also attitudes towards care (see **Box 3**). In this perspective the step towards online care is likely to be smaller for the Finnish nurses. Last, the differences in the ages of the patient populations may also have influenced our findings, since the nurses might see more potential for eHealth applications in younger patients, who they might regard as being more autonomous in their health behaviours.

For peer review only - http://bmjopen.bmj.com/site/about/guidelines.xhtml

Our results concerning dementia prevention are very preliminary, but of special interest. The Finnish nurses liked the idea of including cognitive health as a goal for cardiovascular preventive care, as dementia was regarded as a growing public health problem, and a combined approach could increase people's motivation to engage in behaviour change. However, the nurses felt they could not provide proper support, given their limited knowledge and training on one hand, and the limited extent of conclusive scientific evidence on the other.

Strengths and limitations

The HATICE project is novel in its aim to develop a generic innovative cardiovascular prevention strategy for older people that can be used across European healthcare systems, especially since it involves eHealth. Our qualitative research design enabled us to use the best practices of nurse experts in 'traditional' face-to-face cardiovascular preventive care to make recommendations for optimal health behaviour change support through novel internet-platforms. In qualitative research, international joint analyses are not common because of language barriers. To overcome these, we put much effort into the alignment of our research methodology. The frequent interactions and extensive meetings of the research teams enabled us to explore our findings in the context of the local health care systems. Following grounded theory methodology [25], we deliberately selected nurses that we regarded experts in preventive cardiovascular care. Our research also has some limitations that may have influenced our findings. Information on non-participation was limited. The Finnish nurses had, on average, more years of clinical experience with CV prevention than the Dutch nurses. The patient populations of the Finnish and Dutch nurses were not identical with respect to age. Both of these factors may have influenced our findings. However, since the clinical experience of both groups was very similar, and both countries have similar aims for cardiovascular prevention, we deem the selection of these nurses appropriate for our research purpose. Since we only performed two focus groups, we cannot exclude that a wider range of views could have been collected. For example, one might expect that themes related to training and education requirements would have emerged more prominently from the discussions, but this issue only was mentioned with regard to cognitive health. A further limitation is that cognitive health was only discussed with the Finnish nurses. This issue should be elaborated further in future studies. The striking similarities in the principal themes found in both countries and the consistency of our findings with previous literature mitigates fears that our samples were too limited. Last, when reviewing a summary of our findings, the nurses confirmed that their experiences and views were reflected and did not add new ones, emphasising that the most relevant themes were captured.

Comparison with existing literature

The importance of a relationship of trust, clarifying patients expectations and providing personally tailored support where also main themes in other European qualitative studies on cardiovascular

For peer review only - http://bmjopen.bmj.com/site/about/guidelines.xhtml

preventive care with nurses or patients [28-32]. The positive attitude of the Finnish nurses on self-management of medical issues was consistent with another Finnish study about nurses' and physicians' perceptions of patients' responsibilities in self-care [33]. The reserved attitude of the Dutch nurses was also reflected in a survey among Dutch healthcare professionals, where 50% feared that patients' direct access to their medical record would cause misunderstandings and unnecessary anxiety[34]. A recent qualitative systematic review on nurses' experiences of facilitators and barriers of using telehealth also reported both positive and negative attitudes of nurses towards telehealth. With regard to the nurse-patient relationship, nurses mentioned, on the positive side, that telehealth could improve trusting relationships and lower access to care. On the negative side, nurses reported that telehealth could lead to a loss of human contact. Differences in attitudes were not linked to local health care cultures[35]. Finally, the conviction of all nurses in our study that a coach was essential to complement the internet-platform, is supported by a meta-analysis we performed showing that internet-interventions combined with human support were more effective than 'stand-alone' interventions [36].

Implications for practice

Finnish and Dutch nurses have similar experiences with and views on supporting behaviour change for cardiovascular prevention, but use different practical approaches towards their patients. Including the maintenance of cognitive health as a goal of cardiovascular prevention can provide novel opportunities to frame health behaviour change for both prevention of dementia and CVD, and might augment people's motivation for prevention, but this suggestion should be studied further. The nurses' experiences provide valuable directions for shaping online support in internet-platforms for cardiovascular self-management. This study also indicates that, when introducing new forms of preventive healthcare that involve patient self-management, like internet-platforms, local healthcare practices are to be taken into account to achieve optimal engagement.

For peer review only

ADDITIONAL INFORMATION

Contributors

Study design: CB, ER, EMvC, HS, FM and MK. Data acquisition: CB, MB and AR. Data analysis: CB, UA, MB and AR. Interpretation of results: CB, UA, MB, AR, FM, EMvC and JP. Drafting of the manuscript: CB. Critical revision of the manuscript for important intellectual content: all authors.

Funding

The research leading to these results has received funding from the European Union Seventh Framework Programme for the HATICE-project (FP7/2007-2013, grant agreement n° 305374) and the Multimodal preventive trials for Alzheimer´s Disease: towards multinational strategies – programme (MIND-AD: MIND-AD Academy of Finland (grant agreement n°291803), MIND-AD VTR, Kuopio University Hospital ((grant agreement n°5772815) and MIND-AD Academy of Sweden (Swedish Research Council, 529-2014-7503)) .

Competing interests

The authors have declared no competing interests.

Ethical approval

In the Netherlands, the study was presented to the medical ethics committee of the Academic Medical Centre in the Netherlands and a waiver was provided. In Finland, neither application for ethical approval nor a waiver was required. Written informed consent was obtained from all participants in both countries.

Data sharing statement

Data will not be made publicly available. Please contact the corresponding author for more information.

Acknowledgements

We thank all Finnish and Dutch nurses for their participation in the study, Suzanne Ligthart, Carin Miedema, Paulien Vermunt, Floor Rooskens (respectively discussion moderator, assistant-moderator and assistants in transcription and coding for the Dutch meetings), Lotta Salo, Ejja Pietilä (organisation and summary notes of the Finnish meeting) and Nicola Coley (review of written English) for their assistance to the study.

REFERENCES

1. Oliver D. 21st century health services for an ageing population: 10 challenges for general practice. *The British journal of general practice : the journal of the Royal College of General Practitioners* 2012;62(601):396-7. doi: 10.3399/bjgp12X653435 [published Online First: 2012/08/08]
2. Wu YT, Fratiglioni L, Matthews FE, et al. Dementia in western Europe: epidemiological evidence and implications for policy making. *The Lancet Neurology* 2016;15(1):116-24. doi: 10.1016/s1474-4422(15)00092-7 [published Online First: 2015/08/25]
3. de Bruijn RF, Bos MJ, Portegies ML, et al. The potential for prevention of dementia across two decades: the prospective, population-based Rotterdam Study. *BMC medicine* 2015;13:132. doi: 10.1186/s12916-015-0377-5 [published Online First: 2015/07/22]
4. Kivipelto M, Ngandu T, Laatikainen T, et al. Risk score for the prediction of dementia risk in 20 years among middle aged people: a longitudinal, population-based study. *The Lancet Neurology* 2006;5(9):735-41. doi: 10.1016/s1474-4422(06)70537-3 [published Online First: 2006/08/18]
5. Norton S, Matthews FE, Barnes DE, et al. Potential for primary prevention of Alzheimer's disease: an analysis of population-based data. *The Lancet Neurology* 2014;13(8):788-94. doi: 10.1016/s1474-4422(14)70136-x [published Online First: 2014/07/18]
6. Peters R, Beckett N, Forette F, et al. Incident dementia and blood pressure lowering in the Hypertension in the Very Elderly Trial cognitive function assessment (HYVET-COG): a double-blind, placebo controlled trial. *The Lancet Neurology* 2008;7(8):683-9. doi: 10.1016/s1474-4422(08)70143-1 [published Online First: 2008/07/11]
7. Leventhal H, Weinman J, Leventhal EA, et al. Health psychology: the search for pathways between behavior and health. *Annu Rev Psychol* 2008;59:477-505.
8. Rosenstock IM. The Health Belief Model and Preventive Health Behavior. *Health Education Monographs* 1974;2(4):354-86. doi: 10.1177/109019817400200405
9. Ajzen I. From Intentions to Actions: A Theory of Planned Behavior. In: Kuhl J, Beckmann J, eds. Action Control: From Cognition to Behavior. Berlin, Heidelberg: Springer Berlin Heidelberg 1985:11-39.
10. Bandura A. Self-efficacy: Toward a unifying theory of behavioral change. *Psychological Review* 1977;84(2):191-215. doi: 10.1037/0033-295X.84.2.191
11. Prochaska JO, DiClemente CC, Norcross JC. In search of how people change. Applications to addictive behaviors. *The American psychologist* 1992;47(9):1102-14. [published Online First: 1992/09/01]
12. Weinstein ND. The precaution adoption process. *Health Psychology* 1988;7(4):355-86. doi: 10.1037/0278-6133.7.4.355
13. Burke LE, Dunbar-Jacob JM, Hill MN. Compliance with cardiovascular disease prevention strategies: a review of the research. *Ann Behav Med* 1997;19(3):239-63.
14. Abraham C, Kelly MP, West R, et al. The UK national institute for health and clinical excellence public health guidance on behaviour change: A brief introduction. *Psychology, Health & Medicine* 2009;14(1):1-8. doi: 10.1080/13548500802537903
15. Busse R, Blümel M, Scheller-Kreinsen D, et al. Tackling chronic disease in Europe Strategies, interventions and challenges. *World Health Organization 2010, on behalf of the European Observatory on Health Systems and Policies* 2010;20
16. Bodenheimer TL, K.; Holman, H.; Grumbach, K. Patient Self-Management of Chronic Disease in Primary Care. *Jama* 2002;288(19):2469 - 75.
17. Lorig K, Holman H. Self-Management Education: History, Definition, Outcomes, and Mechanisms. *Ann Behav Med* 2003;26(1):1-7.
18. Griffiths F, Lindenmeyer A, Powell J, et al. Why are health care interventions delivered over the internet? A systematic review of the published literature. *Journal of medical Internet research* 2006;8(2):e10. doi: 10.2196/jmir.8.2.e10 [published Online First: 2006/07/27]
19. Bennett GG, Glasgow RE. The delivery of public health interventions via the Internet: actualizing their potential. *Annual review of public health* 2009;30:273-92. doi: 10.1146/annurev.publhealth.031308.100235 [published Online First: 2009/03/20]

For peer review only - http://bmjopen.bmj.com/site/about/guidelines.xhtml

20. Patnode CD, Evans CV, Senger CA, et al. U.S. Preventive Services Task Force Evidence Syntheses, formerly Systematic Evidence Reviews. Behavioral Counseling to Promote a Healthful Diet and Physical Activity for Cardiovascular Disease Prevention in Adults Without Known Cardiovascular Disease Risk Factors: Updated Systematic Review for the US Preventive Services Task Force. Rockville (MD): Agency for Healthcare Research and Quality (US) 2017.
21. Richard E, Jongstra S, Soininen H, et al. Healthy Ageing Through Internet Counselling in the Elderly: the HATICE randomised controlled trial for the prevention of cardiovascular disease and cognitive impairment. *BMJ Open* 2016;6(6):e010806. doi: 10.1136/bmjopen-2015-010806 [published Online First: 2016/06/12]
22. Glaser BG, Strauss AL. The discovery of grounded theory. *Aldine, Chicago* 1967
23. Strauss A, Corbin J. Grounded theory methodology. *Handbook of qualitative research* 1994:273-85.
24. Tong A, Sainsbury P, Craig J. Consolidated criteria for reporting qualitative research (COREQ): a 32-item checklist for interviews and focus groups. *International Journal for Quality in Health Care* 2007;19(6):349-57. doi: 10.1093/intqhc/mzm042
25. Foley G, Timonen V. Using Grounded Theory Method to Capture and Analyze Health Care Experiences. *Health Services Research* 2015;50(4):1195-210. doi: 10.1111/1475-6773.12275
26. Jongstra S, Beishuizen C, Andrieu S, et al. Development and Validation of an Interactive Internet Platform for Older People: The Healthy Ageing Through Internet Counselling in the Elderly Study. *Telemedicine journal and e-health : the official journal of the American Telemedicine Association* 2016 doi: 10.1089/tmj.2016.0066 [published Online First: 2016/07/28]
27. Puska P, Nissinen A, Tuomilehto J, et al. The community-based strategy to prevent coronary heart disease: conclusions from the ten years of the North Karelia project. *Annual review of public health* 1985;6:147-93. doi: 10.1146/annurev.pu.06.050185.001051 [published Online First: 1985/01/01]
28. Loon MS, van Dijk-de Vries A, van der Weijden T, et al. Ethical issues in cardiovascular risk management: Patients need nurses' support. *Nursing ethics* 2014;21(5):540-53. doi: 10.1177/0969733013505313 [published Online First: 2013/11/22]
29. Ligthart SA, van den Eerenbeemt KD, Pols J, et al. Perspectives of older people engaging in nurse-led cardiovascular prevention programmes: a qualitative study in primary care in the Netherlands. *The British journal of general practice : the journal of the Royal College of General Practitioners* 2015;65(630):e41-8. doi: 10.3399/bjgp15X683149 [published Online First: 2014/12/31]
30. Brobeck E, Bergh H, Odencrants S, et al. Primary healthcare nurses' experiences with motivational interviewing in health promotion practice. *Journal of clinical nursing* 2011;20(23-24):3322-30. doi: 10.1111/j.1365-2702.2011.03874.x [published Online First: 2011/10/25]
31. Boase S, Mason D, Sutton S, et al. Tinkering and tailoring individual consultations: how practice nurses try to make cardiovascular risk communication meaningful. *Journal of clinical nursing* 2012;21(17-18):2590-8. doi: 10.1111/j.1365-2702.2012.04167.x [published Online First: 2012/07/14]
32. Hornsten A, Lindahl K, Persson K, et al. Strategies in health-promoting dialogues--primary healthcare nurses' perspectives--a qualitative study. *Scandinavian journal of caring sciences* 2014;28(2):235-44. doi: 10.1111/scs.12045 [published Online First: 2013/04/19]
33. Jallinoja P, Absetz P, Kuronen R, et al. The dilemma of patient responsibility for lifestyle change: Perceptions among primary care physicians and nurses. *Scand J Prim Health Care* 2007;25(4):244-49. doi: 10.1080/02813430701691778
34. Krijgsman J, Peeters J, Burghouts A, et al. Between the spark and the flame: 2015 eHealth monitor. *Nictiz and the NIVEL Institute [Dutch Institute for Research in the Healthcare Sector]* 2015;October
35. Koivunen M, Saranto K. Nursing professionals' experiences of the facilitators and barriers to the use of telehealth applications: a systematic review of qualitative studies. *Scandinavian journal of caring sciences* 2018;32(1):24-44. doi: 10.1111/scs.12445 [published Online First: 2017/08/05]

For peer review only - http://bmjopen.bmj.com/site/about/guidelines.xhtml

36. Beishuizen CR, Stephan BC, van Gool WA, et al. Web-Based Interventions Targeting Cardiovascular Risk Factors in Middle-Aged and Older People: A Systematic Review and Meta-Analysis. *Journal of medical Internet research* 2016;18(3):e55. doi: 10.2196/jmir.5218 [published Online First: 2016/03/13]
37. Hämäläinen P, Reponen J, Winblad I. eHealth of Finland - Checkpoint 2008. 2009
38. Säkkinen S, Heino A, Häkkinen P, et al. Statistical yearbook on social welfare and health care 2015 - National Institute for Health and Welfare. 2016
39. Vuorenkoski L, Mladovsky P, Mossialos E. Finland: Health system review. *Health Systems in Transition* 2008;10(4):1-168.
40. Ministry of Public Health, Welfare and Sport - Healthcare in the Netherlands. 2016
41. Schäfer W, Kroneman M, Boerma W, et al. The Netherlands: Health system review *Health Systems in Transition* 2010;12(1):1-229.
42. Kringos D, Boerma W, Bourgueil Y, et al. The strength of primary care in Europe: an international comparative study. *The British journal of general practice : the journal of the Royal College of General Practitioners* 2013;63(616):e742-e50. doi: 10.3399/bjgp13X674422
43. http://www.kaypahoito.fi/web/english/guidelineabstracts/guideline?id=ccs00014 [Finnish Current Care Guideline on hypertension (2014)]. accessed 7-12-2016 2016.
44. http://www.kaypahoito.fi/web/english/guidelineabstracts/guideline?id=ccs00032 [Finnish Current Care Guideline on diabetes (2016)]. accessed 7-12-2016 2016.
45. http://www.kaypahoito.fi/web/english/guidelineabstracts/guideline?id=ccs00055 [Finnish Current Care Guideline on dyslilidaemias (2013)]. accessed 7-12-2016 2016.
46. Boomsma L, Lakerveld-Heyl K, Gorter K, et al. Landelijke Eerstelijns Samenwerkings Afspraak Diabetes mellitus type 2. *Huisarts Wet* 2006;49(8):418-24.
47. den Boer J. Protocollair CVRM: Nederlands Huisartsen Genootschap 2016.
48. Faber E, De Bont M, Beusmans G, et al. Landelijke Eerstelijns Samenwerkings Afspraak CVA. *Huisarts Wet* 2007;50(3):S5-S8.
49. http://www.kanta.fi/en [Website of the Finnish Patient Data Repository, where Finnish people can access their own medical record]. accessed 7-12-2016 2016.

For peer review only

For peer review only - http://bmjopen.bmj.com/site/about/guidelines.xhtml

1 **BOXES**

2

3 **Box 1:** the Finnish and Dutch primary care systems

	Finland	The Netherlands
Organisation	Primary care is delivered through public healthcare centres and through occupational health facilities[37-39]. Companies offer occupational health facilities to their employees, including both preventive and curative health services, delivered through semi-private healthcare centres that work with nurses in a similar fashion to the public centres.	General practices or small health care centres. In most general practices, continuity of care is ensured by allocating the patient to one GP.
Main focus	Primary care has a strong position and important gatekeeper function. Health promotion and disease prevention have been the main focus of health care policy for decades.	Access to care for everyone and solidarity through medical insurance[40 41]. GPs are gatekeepers of the healthcare system and provide acute, chronic and preventive care.
Accessibility	Often, healthcare centres cover large geographical areas that are sparsely populated and often have staff shortages, contributing to long waiting lists and lack of personal continuity of care. Due to these waiting lists, many employees go to their occupational health service instead.	Since the Netherlands is densely populated, people often live within a short distance of their general practice. Access to GPs is efficient; there are no waiting lists[42].
Role of primary care nurses	Important position: nurses work in close collaboration with GPs and have their own consulting hours to assess patients. Regarding cardiovascular prevention, they monitor patients with diabetes, hypertension and dyslipidaemia, as described in national guidelines[43-45].	Important position: for several decades, GPs have delegated tasks to practice nurses in chronic disease management. Currently, nurses provide a substantial part of cardiovascular risk management care, including diabetes care, following regional and national guidelines and work descriptions[9 46-48].
Patient autonomy and eHealth culture to date	The first European country to introduce a law (in 1993) defining the patient's right to access to all medical information and the right to autonomy (patient's informed consent for any medical treatment). All	Informed consent is ensured by law, but in daily practice, consent is often assumed and only explicitly discussed when treatment options can have far-reaching consequences[41]. Almost all GPs use electronic medical

For peer review only - http://bmjopen.bmj.com/site/about/guidelines.xhtml

	healthcare centres use electronic medical records. A national patient data repository is under development to provide patients complete access to their own electronic medical record[49]	records. Patients have the right to inspect their medical records, but do not have complete access to them.

Box 2. Main topics discussed

Part 1

- Prevention of cardiovascular disease and dementia: attitude and experiences
- Good guidance of behaviour change
- Relationship with the patient

Part 2

- Attitude towards the internet-platform and online-support
- Role and responsibilities of the internet-coach
- Interaction with the patients online

Box 3. Key features of the HATICE internet-platform with coaching

- Patient-centred: the patient can login on to a personal portal to review and manage his/her personal cardiovascular risk profile
- Improving health knowledge: the patient can access educational modules about cardiovascular risk factors and lifestyle
- Goal setting and self-monitoring: the patient can set his/her own goals for behaviour change and monitor how he/she is doing by entering self-measurements or keeping a diary
- Coaching: the coach monitors the patient's self-management and they can communicate online through messages

For peer review only - http://bmjopen.bmj.com/site/about/guidelines.xhtml

1

For peer review only

For peer review only - http://bmjopen.bmj.com/site/about/guidelines.xhtml

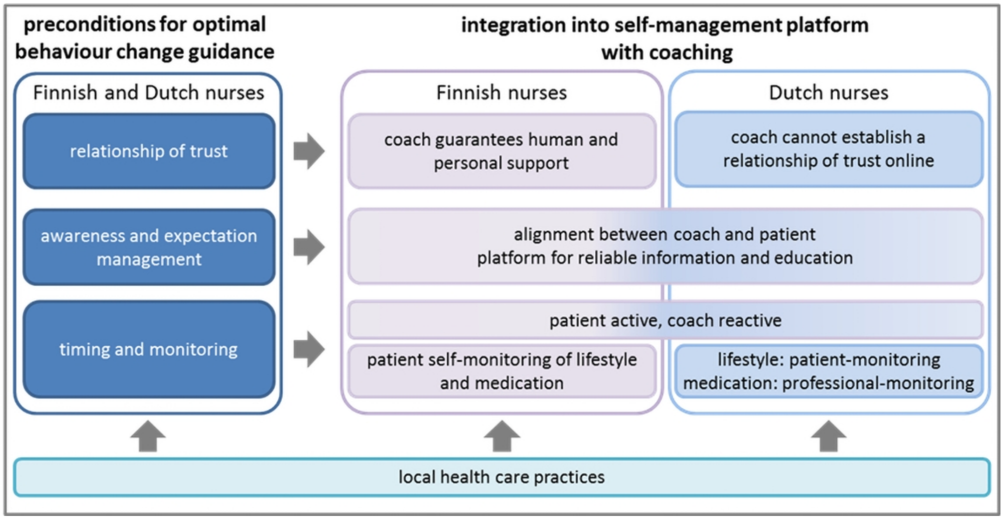

Caption: Figure 1. Schematic visualization of the main themes and their connections
Legend: Left, the three main preconditions for good behaviour change guidance in cardiovascular preventive care that both Finnish and Dutch nurses identified, are depicted. Right of this, it is shown how the Finnish and Dutch nurses suggest to realise these preconditions in the online setting. Since there were differences between the nurses this is depicted separately for the Finnish and Dutch nurses. Below it is shown that local health practices influenced both the preconditions (and their operationalization (not shown in figure but explained in results section)) and the integration into online support.

194x99mm (300 x 300 DPI)

APPENDIX 1 to 'Integrating nurses' experiences with supporting behaviour change for cardiovascular prevention into a self-management internet-platform in Finland and the Netherlands: a qualitative study'

Appendix 1: COREQ checklist

**No.**	**Item**	**Explanation**	**Reported on page no.**
Domain 1:Research team and reflexivity			
1	Interviewers	NL: Pols AJ and Ligthart S<br>FI: Rosenberg A	Not reported in manuscript
2	Credentials of the researchers involved in data collection and analysis	NL: Beishuizen CRL: MD PhD-student; Rooskens F: BSc; Lighthart S: MD, PhD-student; Pols AJ: prof; Moll van Charante EP: MD PhD<br>FI: Akenine U: PhD-student; Barbera M: PhD; Rosenberg A: PhD-student	Not reported in manuscript
3	Occupation of the researchers involved in data collection and analysis	NL: Beishuizen CRL: PhD-student; Rooskens F: medical student; Ligthart S: general practitioner in training, PhD-student; Pols AJ: professor in medical ethics; Moll van Charante EP: postdoc researcher, general practitioner<br>FI: Akenine U: PhD-student, research nurse; Barbera M: postdoc researcher; Rosenberg A: PhD-student	Not reported in manuscript
4	Gender	All researchers are female, except for Moll van Charante EP, who is male	Not reported in manuscript
5	Experience and training	See main text	p5
6	Relationship established	NL: there was an indirect relationship between research team and the partcipants, because the nurses had been involved in a previous research project of the research team in which 2 researchers (Ligthart S and Moll van Charante EP) were also involved<br>FI: no previous relationship established	Not reported in manuscript
7	Partcipant knowledge of the	NL: participants knew the professional background of the moderators	Not reported in

For peer review only - http://bmjopen.bmj.com/site/about/guidelines.xhtml

	interviewer	FI: participants knew the professional background of the moderator	manuscript
8	Interviewer characteristics	NL: Pols AJ conducts qualitative research on the ethics of use of technology and eHealth in medical care. Ligthart S conducts quantitative and qualitative research on cardiovascular prevention in older people<br><br>FI: Rosenberg A conducts research on prevention of dementia	Not reported in manuscript
Domain 2: Study design			
9	Methodological information and theory	See main text	P5 and p6
10	Sampling	See main text	P5
11	Method of approach	See main text	P5
12	Sample size	See main text	P5
13	Non-participation	See main text	P5
14	Setting of data collection	See main text	P5
15	Presence of non-participants	NL: Eric Moll- van Charante and Pim Happel were present as non-participating audience<br><br>FI: no others were present	Not reported in manuscript
16	Description of sample	See main text	P5 and p6
17	Interview guide	See main text	P6
18	Repeat interviews	Not performed	
19	Audio/visual recording	See main text	P6
20	Field notes	See main text	P6
21	Duration	See main text	P6

For peer review only - http://bmjopen.bmj.com/site/about/guidelines.xhtml

22	Data saturation	See main text	P7 and P15
23	Transcripts returned	Not performed	
Domain 3: analysis and findings			
24	Number of data coders	NL: 2<br>FI: 2	P6
25	Description of the coding tree	See main text (figure 1)	p14
26	Derivation of themes	See main text	P6
27	Software	No special qualitative software was used	
28	Participant checking	See main text	P7 and p15
29	Quotations presented	See main text	P8– p13
30	Data and findings consistent	See main text	P8 – p13
31	Clarity of major themes	See main text and figure 1	P8-p14
32	Clarity of minor themes	Within the groups, the Dutch and Finnish nurses shared opinions and experiences on most topics. Between the Dutch and Finnish groups, some interesting differences in opinions and experiences were identified. We choose therefore to focus on these differences when presenting our results, but not on diverse cases within the groups.<br><br>Minor themes were not discussed due to word limits	Not reported in manuscript

For peer review only - http://bmjopen.bmj.com/site/about/guidelines.xhtml

APPENDIX 2 to 'Integrating nurses' experiences with supporting behaviour change for cardiovascular prevention into a self-management internet-platform in Finland and the Netherlands: a qualitative study'

Appendix 2: Topic list

**FOCUS GROUP nurses part ONE cardiovascular prevention**		
**Overview part one**	**Topic (possible items)**	**Possible questions**
Aim of the meeting is to learn from the nurses' experiences in cardiovascular prevention: what are best practices to stimulate behaviour change, what is their attitude towards a prevention program for older people via the internet, how would they support people in lifestyle change through the internet?		
	**CVD prevention**	
	Activities on CVD prevention	Do you currently conduct activities on CVD prevention?
	Experiences / lessons learned	What are your experiences and lessons learned, especially regarding giving 'medical' guidance versus giving lifestyle guidance and regarding behavior change?
	**Dementia prevention**	In the last decade, we got more and more indications from neurological research that risk factors for cardiovascular disease are also risk factors for dementia. So, possibly, reducing cardiovascular risk may also postpone or prevent dementia.
	Attitude towards dementia prevention	What is your attitude / your ideas regarding dementia prevention?
	Awareness of dementia risk and compliance to lifestyle change	Most people are not yet aware of the association between CV risk and dementia, but do seem to be very afraid of dementia. Do you think that more awareness would enhance compliance/adherence to lifestyle change? What are your ideas on this?
	**Relationship with participants and regular healthcare system**	
	Guiding lifestyle change	Which factors could contribute to good guidance of lifestyle change?
	Relationship with patient	Which factors could contribute to a good relationship with your patient?
	Relation with GP and regular practice assistant	How should the platform coach link with the patients' GP and regular practice assistant/nurse (the regular healthcare system)?

For peer review only - http://bmjopen.bmj.com/site/about/guidelines.xhtml

	Attitude towards internet intervention	What is your attitude towards a prevention program via the internet (with support from a coach)?
		How should the platform coach link with the patients' GP and regular practice assistant/nurse (the regular healthcare system)?
**FOCUS GROUP nurses part TWO The platform**		
Assistant moderator explains about HATICE and platform by showing the powerpoint with screen shots of the platform		
	**Information required for support**	Imagine yourself being a coach using the internet-platform:
		Which kind of information regarding the participants do you need to be able to support them?
	**Role and responsibilities**	
	Responsibility goal setting	Who is responsible for goal setting? (capability of patients)
	Role in lifestyle groups	How do you see your role in creating lifestyle groups and how can participation be encouraged?
	**Interaction with participants**	
	Experience with motivational interviewing	Do you have experience with motivational interviewing, how could this technique be used by the coach?
	Frequency of contact	How often would you like to have contact with your patient?
	Mode of communication	Which mode of communication do you prefer? (phone, skype +/- webcam, email, chat…)
	Network of support	What should be the role of the participant network of support in lifestyle change and how can this network be engaged?
	Alerts / reminders	Do you like to receive automatic alerts/reminders when patients have alarming values or did not log-on?
Assistant moderator and moderator verify key messages from focus group		
Moderator thanks nurses		

For peer review only - http://bmjopen.bmj.com/site/about/guidelines.xhtml
